# Preclinical evidence for luteolin in ulcerative colitis: a meta-analysis and systematic review

**DOI:** 10.3389/fphar.2025.1639644

**Published:** 2025-07-30

**Authors:** Yiyi Feng, Xingyao Lu, Enjia Guo, Jianling Mo, Yichuan Xv

**Affiliations:** ^1^ Department of Traditional Chinese Medicine, Sir Run Run Shaw Hospital, School of Medicine, Zhejiang University, Hangzhou, Zhejiang, China; ^2^ Department of Gastroenterology, Longhua Hospital, Shanghai University of Traditional Chinese Medicine, Shanghai, China; ^3^ Department of Gastroenterology and Hepatology, Hangzhou Red Cross Hospital, Hangzhou, China

**Keywords:** luteolin, ulcerative colitis, systematic review, meta analysis, animal model

## Abstract

**Background:**

Evidence suggests that luteolin (LUT) may offer therapeutic potential in treating ulcerative colitis (UC), though its specific pharmacological mechanisms remain incompletely understood. This meta-analysis aims to assess the pharmacological effects of LUT in UC animal models and investigate its potential mechanisms of action.

**Methods:**

A comprehensive search of five databases, namely, PubMed, Web of Science, Embase, EBSCO, and ScienceDirect, was conducted to identify studies investigating the effects of LUT on UC. The search, covering data up to March 2025, yielded 19 eligible studies involving a total of 327 animal subjects. The outcomes were analyzed using the standard mean difference with a 95% confidence interval in R (4.3.3) software.

**Results:**

The meta-analysis revealed that LUT significantly ameliorated colon length, reduced the disease activity index, alleviated body weight loss, and decreased histological scores. Further mechanistic analysis indicated that LUT exerts its effects through multiple mechanisms, including the reduction of pro-inflammatory cytokines, elevation of anti-inflammatory cytokines, promotion of tight junction protein expression, and improvement of oxidative stress-related indices. However, LUT appears to have no significant impact on the α-diversity of the intestinal microbiota.

**Conclusion:**

This study suggests that LUT may exert significant therapeutic effects in UC animal models through antioxidant, anti-inflammatory, immunomodulatory, and barrier-protective mechanisms. Further clinical studies and translational research are essential to bridge the gap between animal models and human applications.

**Systematic Review Registration:**

https://inplasy.com/inplasy-2025-5-0055/, identifier INPLASY202550055.

## 1 Introduction

Ulcerative colitis (UC) is a chronic, recurrent intestinal inflammatory disease, and its global burden has increased substantially over the past 3 decades, particularly in industrialized countries (Agrawal and Jess, 2022). Immune-mediated inflammation is widely acknowledged as a central factor in the development of UC (Le Berre et al., 2023; Xv et al., 2024). The introduction of biologic agents targeting key inflammatory factors such as interleukin-23 (IL-23) and tumor necrosis factor-α (TNF-α) has improved the management of various degrees of UC (D’Haens and van Deventer, 2021; Feagan et al., 2016). However, around 40% of patients continue to experience either primary or secondary non-response. Additionally, prolonged use of these agents may raise the risk of infections and malignancies. Although immune inflammation is a prominent feature of UC, we must admit that UC is a pathological result driven by multiple factors, including intestinal dysbiosis and mucosal barrier disruption (Foppa et al., 2024; Mansouri et al., 2025). In light of recent discoveries of natural products with promising clinical potential, researchers are increasingly exploring natural compounds with multi-target regulatory effects as complementary therapeutic options for UC (Lu et al., 2025).

Luteolin (LUT), a flavonoid commonly present in medicinal plants such as Chrysanthemum (*Chrysanthemum morifolium*) and Honeysuckle (*Lonicera japonica*), displays various biological effects, such as anti-inflammatory, immunomodulatory, and intestinal microecology-improving effects (Xue et al., 2023; Li B. et al., 2021). Emerging studies have found that applying LUT can significantly alleviate experimental colitis. In further mechanistic exploration, LUT has been shown to regulate inflammation-related pathways (e.g., Nuclear Factor kappa-B (NF-κB) signaling pathway), inhibiting pro-inflammatory cytokines (Li B. et al., 2021; Li et al., 2023). It also promotes the colonization of beneficial bacteria and enhances intestinal epithelial integrity by upregulating the expression of tight junction proteins, including zonula occludens-1 (ZO-1) (Yang et al., 2025a). However, the current body of evidence is limited by several key factors. Firstly, there is significant heterogeneity in research protocols, including variations in animal dosage (ranging from 10 to 100 mg/kg) (Li B.-L. et al., 2021; Magadán-Corpas et al., 2024) and treatment duration (spanning 4–41 days) (Li B.-L. et al., 2021; Xie et al., 2022). Additionally, a substantial translational gap exists, as current studies are confined to experimental colitis models and lack corresponding clinical investigations. Furthermore, systematic evaluation is lacking, with no studies that quantitatively integrate LUT’s overall effects on core UC outcomes, such as the disease activity index (DAI) and histology.

Based on previous research, we hypothesize that LUT exerts significant therapeutic effects on UC models through multi-target mechanisms, but its efficacy requires thorough quantitative integration and mechanistic clarification. This study, through a rigorous literature screening and meta-analysis, offers the first evidence-based integration of preclinical studies on LUT’s effects in the treatment of UC. LUT is recognized as one of the pan-assay interfering compounds (PAINS), which may yield false positive results in *in vitro* experiments due to its PAINS properties (e.g., redox activity, nonspecific binding); therefore, only animal experiments have been involved in this study (Bolz et al., 2021; Magalhães et al., 2021). It quantitatively evaluates LUT’s impact on clinical indicators such as the DAI and colon length (CL) and systematically summarizes its mechanisms. Subgroup analyses are conducted to elucidate the therapeutic characteristics of LUT. The findings aim to provide prioritized recommendations for the design of future clinical trials.

## 2 Methods

This manuscript follows the reporting guidelines outlined in the Preferred Reporting Items for Systematic Reviews and Meta-Analyses (PRISMA) statement (Page et al., 2021). The study was formally registered on INPLASY (https://inplasy.com/; registration number: INPLASY202550055).

### 2.1 Literature search

To collect detailed information on preclinical animal studies regarding the use of LUT for treating UC, we performed a literature search across five databases: PubMed, Web of Science, Embase, EBSCO, and ScienceDirect. The search was conducted until March 5, 2025. A comprehensive search strategy was implemented to ensure thorough coverage of relevant literature. After consulting with all authors, medical subject headings terms and free-text search terms were combined to identify diseases and drug interventions, including “ulcerative colitis,” “luteolin,” and “3′,4′,5,7-Tetrahydroxyflavone” (Supplementary Table S1).

### 2.2 Inclusion criteria

Every animal study assessing the efficacy of LUT in UC treatment was considered for inclusion, regardless of the animal species, modeling methods, sex, or sample size. The studies selected met the following criteria: (Agrawal and Jess, 2022): published research articles about animal studies; (Le Berre et al., 2023); studies with independent intervention and model groups; (Xv et al., 2024); studies with any dose, route, method, and regimen of administration; (D’Haens and van Deventer, 2021); intervention group received only LUT treatment, while model group did not receive any treatment or was treated with vehicle; (Feagan et al., 2016); availability of experimental data.

### 2.3 Exclusion criteria

The following studies were excluded: (Agrawal and Jess, 2022): clinical studies, *in vitro* experiments (*in vitro* experiments were strictly excluded from this study considering that LUT is one of the PAINS (Bolz et al., 2021; Magalhães et al., 2021)), reviews, and case reports; (Le Berre et al., 2023); studies with significant data bias; (Xv et al., 2024); duplicate publications; (D’Haens and van Deventer, 2021); studies with missing experimental data; (Feagan et al., 2016); studies not reporting any of the primary outcomes prespecified in our protocol.

### 2.4 Data extraction

The following data were extracted independently by two authors: (Agrawal and Jess, 2022): the first author and the publication year (if the same author appeared in multiple studies, different letters (a, b) were used to distinguish them); (Le Berre et al., 2023); animal species, sex, and sample size; (Xv et al., 2024); modeling method for UC in animals; (D’Haens and van Deventer, 2021); intervention measures, including the intervention period and dosage for both the model and treatment groups; (Feagan et al., 2016); outcome measures, including histological score (HSC), body weight change (BWC), CL, DAI, IL-6, IL-1β, IL-17, IL-10, TNF-α, ZO-1, occludin, myeloperoxidase (MPO), Superoxide dismutase (SOD) and malondialdehyde (MDA). Notably, for primary outcome HSC, all included studies used a standardized scoring system to assess tissue damage, which consists of five components, including the severity of inflammation, degree of inflammatory cell infiltration, epithelial damage, extent of lesions, and edema score, each evaluated on a scale from 0 to 3 (Vukelić et al., 2020). For each animal’s colon section, the most representative area of the lesion was selected for evaluation at 200x magnification. It integrates the infiltration degree of inflammatory cells and the damage of mucosal structure to make a systematic assessment of intestinal inflammation is suitable for the evaluation of all colitis models. In cases where the treatment group involved multiple dosage levels, the highest dosage group was selected for meta-analysis. For data presented in the figures, the original data were initially obtained from the authors; otherwise, the graph data were quantified using Origin software.

### 2.5 Quality assessment

The risk of bias was independently evaluated by two authors utilizing the 10-item Scale for the Assessment of Risk in Laboratory Animal Experiments (SYRCLE) (Hooijmans et al., 2014). The quality assessment criteria encompassed selection bias, performance bias, detection bias, attrition bias, reporting bias, and additional sources of bias. Any discrepancies during the assessment process would be addressed by consultation with the corresponding author.

### 2.6 Data analysis

The standardized mean difference (SMD) and 95% confidence intervals (CIs) were calculated to assess the LUT’s effects on outcomes, as all outcomes were continuous indices. A *p*-value of 0.05 was set as the significance threshold for comparing the LUT intervention with the model groups. Heterogeneity was evaluated using the I^2^ statistic. An I^2^ statistic ≤50% indicated mild heterogeneity, so we used the fixed-effect model to conduct the meta-analysis. If I^2^ >50%, the random-effects model was applied because of significant heterogeneity. Subgroup analyses were conducted to explore sources of heterogeneity. According to different animal species, modeling methods, dosage levels, and treatment duration of included studies, we divided them into several subgroups and repeated the same analyses. For each outcome, if the number of included studies exceeded 10, the Egger test was employed to assess publication bias. A *p*-value of less than 0.05 was considered indicative of significant publication bias, which would be corrected using the trim-and-fill method. This method identified the missing studies (those that would have been published if there were no bias) and then imputed their effect sizes to generate a more accurate estimate of the overall effect. The procedure adjusts the funnel plot and recalculates the pooled effect size, providing a bias-corrected estimate. Sensitivity analysis was conducted to evaluate the impact of each study on the overall results. The above analyses were conducted using R (4.3.3) software.

## 3 Results

### 3.1 Literature search and screening

**FIGURE 1:**
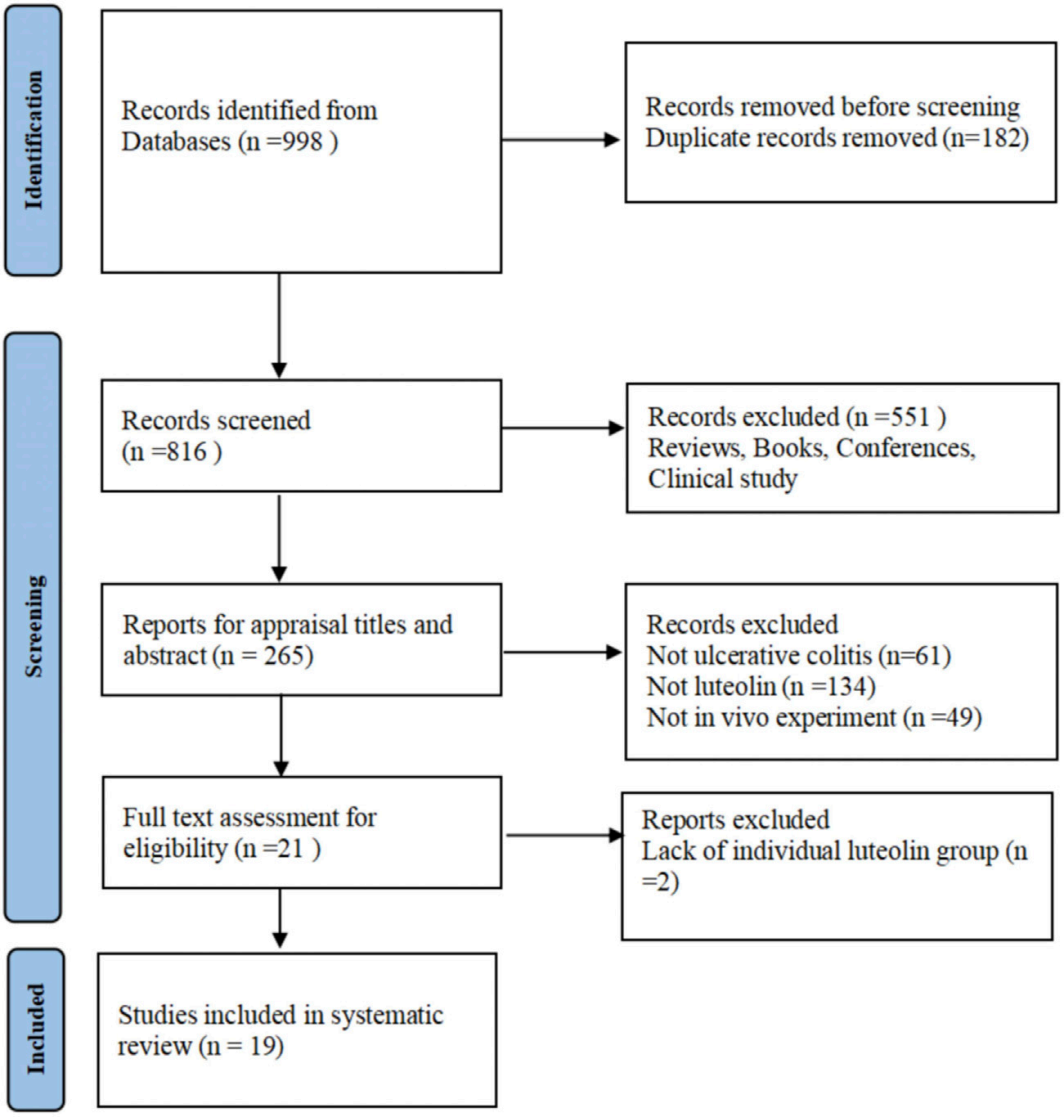
Flowchart of the literature search and screening process.

Following the search strategy, 998 English articles were obtained through preliminary search, including 45 from PubMed, 116 from Embase, 65 from Web of Science, 57 from EBSCO, and 715 from ScienceDirect. After importing into EndNote software and removing duplicates, 816 articles remained. Among the remaining articles, 797 were deleted after assessing the title, abstract, and full text. Finally, 19 articles were included after full-text evaluation (Xue et al., 2023; Li B. et al., 2021; Li et al., 2023; Yang et al., 2025a; Li B.-L. et al., 2021; Magadán-Corpas et al., 2024; Xie et al., 2022; Vukelić et al., 2020; Li et al., 2016; Liu et al., 2025; Xie et al., 2024; Yang et al., 2025b; Liu et al., 2020; Suga et al., 2021; Tan et al., 2024; Bi et al., 2024; Tan et al., 2022; Pan et al., 2025; Lu et al., 2023). The process of literature search and screening was demonstrated in Figure 1. Most of the encompassed studies were published in the past 5 years, indicating that LUT is an emerging natural product with therapeutic effects on UC and may have translational potential.

### 3.2 Features of the included studies

This study encompassed 19 articles involving 327 animals. Within this combined cohort, 161 animals were allocated to the intervention group while 166 animals were allocated to the model group. The animal species used included 52 BALB/c mice (Yang et al., 2025a; Yang et al., 2025b; Tan et al., 2024), 175 C57BL/6 mice (Xue et al., 2023; Li B.-L. et al., 2021; Xie et al., 2022; Vukelić et al., 2020; Li et al., 2016; Liu et al., 2025; Xie et al., 2024; Suga et al., 2021; Bi et al., 2024; Pan et al., 2025), 12 Sprague-Dawley rats (Lu et al., 2023), 56 Wistar rats (Li B. et al., 2021; Li et al., 2023; Liu et al., 2020), 16 Fischer 344 rats (Magadán-Corpas et al., 2024), and 16 Kunming mice (Tan et al., 2022). In terms of sex specificity, 17 studies used only male animals (Xue et al., 2023; Li B. et al., 2021; Li et al., 2023; Yang et al., 2025a; Li B.-L. et al., 2021; Magadán-Corpas et al., 2024; Xie et al., 2022; Vukelić et al., 2020; Li et al., 2016; Xie et al., 2024; Yang et al., 2025b; Liu et al., 2020; Suga et al., 2021; Tan et al., 2024; Bi et al., 2024; Tan et al., 2022; Lu et al., 2023), one study focused exclusively on female animals (Pan et al., 2025), and one study failed to specify the sex of animals (Vukelić et al., 2020). The administered doses of LUT ranged from 10 mg/kg to 100 mg/kg. The majority of studies employed oral gavage for administration (Xue et al., 2023; Li B. et al., 2021; Li et al., 2023; Yang et al., 2025a; Li B.-L. et al., 2021; Xie et al., 2022; Vukelić et al., 2020; Li et al., 2016; Liu et al., 2025; Xie et al., 2024; Yang et al., 2025b; Liu et al., 2020; Tan et al., 2024; Bi et al., 2024; Tan et al., 2022; Pan et al., 2025; Lu et al., 2023), one study utilized intraperitoneal injection (Magadán-Corpas et al., 2024), and one study incorporated the treatment into the feed (Suga et al., 2021). The intervention durations varied between 4 and 41 days, with ten studies involving long-term administration (lasting ≥14 days) (Xue et al., 2023; Li B. et al., 2021; Li et al., 2023; Yang et al., 2025a; Xie et al., 2022; Xie et al., 2024; Yang et al., 2025b; Suga et al., 2021; Bi et al., 2024; Lu et al., 2023) and nine studies involving short-term administration (<14 days) (Li B.-L. et al., 2021; Magadán-Corpas et al., 2024; Vukelić et al., 2020; Li et al., 2016; Liu et al., 2025; Liu et al., 2020; Tan et al., 2024; Tan et al., 2022; Pan et al., 2025). In this study, the experimental group received LUT treatment, while the model group received a solvent or blank control. For UC induction, two studies used 2,4,6-Trinitrobenzenesulfonic acid (TNBS) (Liu et al., 2020; Lu et al., 2023) enema, and 17 studies added dextran sulfate sodium (DSS) to drinking water (Xue et al., 2023; Li B. et al., 2021; Li et al., 2023; Yang et al., 2025a; Li B.-L. et al., 2021; Magadán-Corpas et al., 2024; Xie et al., 2022; Vukelić et al., 2020; Li et al., 2016; Liu et al., 2025; Xie et al., 2024; Yang et al., 2025b; Suga et al., 2021; Tan et al., 2024; Bi et al., 2024; Tan et al., 2022; Pan et al., 2025).

The literature included was compiled to assess the impact of LUT on UC. Sixteen studies measured DAI, fifteen assessed CL, eleven assessed BWC, and eleven performed histopathological analysis. Regarding inflammatory biomarkers, four studies reported IL-10 levels, two studies assessed IL-17 levels, three studies evaluated C-reactive protein (CRP) levels, eight studies assessed TNF-α, and seven studies measured IL-1β levels and IL-6 levels. Regarding oxidative stress-related indicators, five studies measured MPO activity, three studies reported MDA levels, and four studies assessed SOD levels. Regarding mucosal barrier-related indicators, five studies analyzed ZO-1 levels, and four studies measured occludin levels. Additionally, five studies evaluated the Chao index and Shannon index. Detailed information from the encompassed studies was provided in Table 1.

**TABLE 1:** Basic characteristics of the included studies.

Study (year)	Species (sex, n = Lut/model group, weight)	Model method	Lut group (administration, drug dose, duration)	Model group (administration, drug dose, duration)	Outcome index
Xue et al. (2023)	C57BL/6 mice (male, 8/8)	3% DSS in the drinking water (7 days)	By gavage, 50 mg/kg, 14 days	By gavage, 0.5% CMC-Na, 14 days	②③④
Li B. et al. (2021)	Wistar rats (male, 10/10, 200 ± 20 g)	3.5% DSS in the drinking water (10 days)	By gavage, 34.6 mg/kg, 14 days	By gavage, 0.9% sodium chloride, 14 days	②⑯⑰
Li et al. (2023)	Wistar rats (male, 10/10, 200 ± 20 g)	3.5% DSS in the drinking water (10 days)	By gavage, 34.6 mg/kg, 14 days	By gavage, normal sodium chloride, 14 days	①⑧⑨
Yang et al. (2025a)	BALB/c mice (male,8/8,18–22 g)	3% DSS in the drinking water (7 days)	By gavage, 25 mg/kg, 14 days	By gavage, 0.5%CMC-Na,14 days	①②③④⑤⑥⑦⑧⑭⑮⑯⑰
Li B.-L. et al. (2021)	C57BL/6 mice (male, 8/8)	4% DSS in the drinking water (7 days)	By gavage, 100 mg/kg, 4 days	Not mention	②③④⑭
Magadán-Corpas et al. (2024)	Fischer 344 rats (male, 8/8)	3% DSS in the drinking water (7 days)	Intraperitoneal, 10 mg/kg, 11 days	Intraperitoneal, PBS, 11 days	①②③⑥⑦⑪⑯⑰
Xie et al. (2022)	C57BL/6J mice (male, 12/12, 21–22 g)	2 %DSS for the 5 days, sterile distilled water for 4 days, continue 4 cycles	By gavage, max20 mg/kg, 41 days	Not mention	①②③④⑥⑦⑧⑩⑭⑮
Vukelić et al. (2020)	C57BL/6 mice (male, 6–8/6–8)	3% DSS in the drinking water (5–7 days)	By gavage, 100 mg/kg, 5 days	Not mention	③④
Li et al. (2016)	C57BL/6 mice (male, 6/6)	3% DSS in the drinking water (7 days)	By gavage, 50 mg/kg, 7 days	By gavage, 0.5% carboxymethyl cellulose, 7 days	②③④⑫⑬
Liu et al. (2025)	C57BL/6J mice (10/10, 23–25 g)	2.5% DSS in the drinking water (7 days)	By gavage, 100 mg/kg, 7 days	Not mention	①②③④
Xie et al. (2024)	C57BL/6J mice (male, 12/12, 20–22 g)	2% DSS in the drinking water for 5 days, distilled water for 4 days, a total of four rounds	By gavage, 10 mg/kg, 31 days	By gavage, 0.5% CMC-Na, 31 days	②③④⑭⑮
Yang et al. (2025b)	BALB/c mice(male, 10/10, 18–22 g)	3% DSS in the drinking water (7 days)	By gavage,50 mg/kg, 17 days	By gavage, 0.5% CMC-Na, 17 days	①②③④⑤⑥⑦⑧⑭⑮
Liu et al. (2020)	Wistar rats (male, 8/8, 180–200 g	Enema with 2.5% TNBS for 3 days	By gavage, 100 mg/kg, 10 days	Not mention	⑥⑧⑨⑪⑫⑬
Suga et al. (2021)	C57BL/6N mice (male, 5/10)	2.5% DSS in the drinking water (7 days)	By diet, 80 mg/kg, 14 days	Not mention	②③⑪
Tan et al. (2024)	BALB/c mice (male, 8/8, 16–20 g)	3% DSS in the drinking water(10 days)	By gavage, 100 mg/kg, 10 days	Not mention	①②③⑥⑧⑯⑰
Bi et al. (2024)	C57BL/6 mice (male, 12/12)	3% DSS in the drinking water(7 days)	By gavage, 5 mg/kg, 14 days	Not mention	①②③⑯⑰
Tan et al. (2022)	Kunming mice (male, NA/NA, 22–25 g)	3% DSS in the drinking water(7 days)	By gavage, 20 mg/kg, 7 days	Not mention	①②③④⑤⑦⑧⑩⑪
Pan et al. (2025)	C57BL/6J (mice, female, 4/4, 18–20 g)	3% DSS in the drinking water(7 days)	By gavage, 17 mg/kg, 5 days	Not mention	①②③④⑦⑪⑬
Lu et al. (2023)	Sprague Dawley rats (male, 6/6, 180–220 g)	Enema with 2.5% TNBS for	By gavage,100 mg/kg, 28 days	Physiological saline, 28 days	①②⑤⑥⑦⑧⑨⑫⑬

Notes: ①BWC ②DAI ③CL ④HSC ⑤IL-10 ⑥IL-1β ⑦IL-6 ⑧TNF-α ⑨CRP ⑩IL-17 ⑪MPO ⑫MDA ⑬SOD ⑭ZO-1 ⑮occludin ⑯Chao ⑰Shannon.

### 3.3 Study quality

**FIGURE 2:**
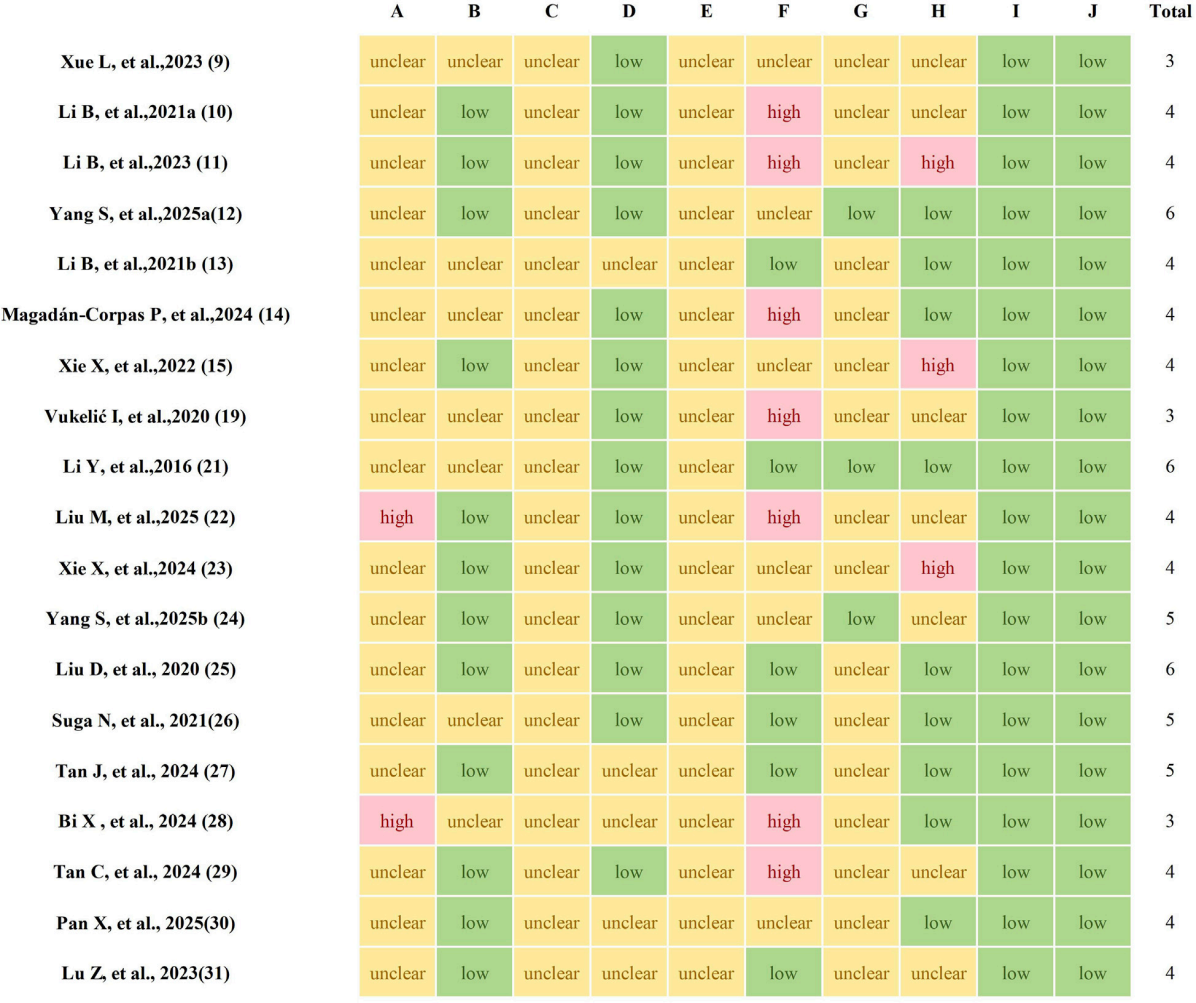
The methodological quality of included studies. A, Sequence generation; B, Baseline characteristics; C, Allocation concealment; D, Random housing; E, Blinding of experimentalists; F, Random outcome assessment; G, Blinding of outcome assessors; H, Incomplete outcome data; I, Selective outcome reporting; J, Other sources of bias.

The quality of the studies was evaluated using a 10-item scale, scoring from 3 to 6 points. The distribution of scores was as follows. Of the 19 studies reviewed, 17 mentioned random allocation of animals but did not provide details about the specific randomization method used. The remaining four studies did not report any form of randomization of the animals. Housing conditions were identical in 14 studies, whereas three studies did not report this. Three studies revealed the existence of incomplete data, while 16 studies confirmed the availability of complete data. No cases of selective reporting were found. None provided precise details regarding the blinding of participants and personnel. However, three studies reported blinding of outcome assessors. Furthermore, none of the studies reported allocation concealment. Finally, potential sources of bias were not identified in any of the studies. The methodological quality of the incorporated studies was detailed in Figure 2.

### 3.4 Results of the meta-analysis

#### 3.4.1 Histopathology and clinical indices

**FIGURE 3:**
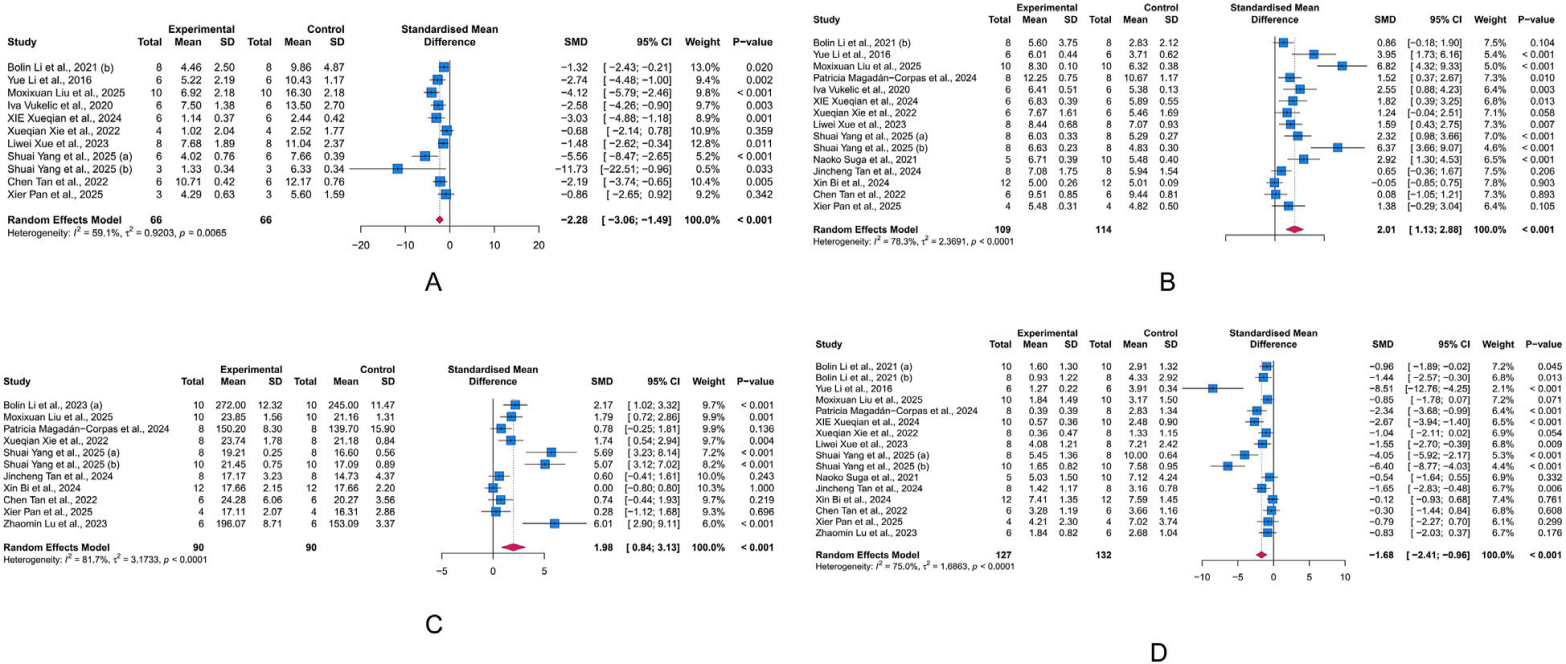
Forest plots showing results of histological and primary outcomes. **(A)** Histological score (HSC). **(B)** Colon length (CL). **(C)** Body weight change (BWC). **(D)** Disease activity index (DAI). The blue squares and horizontal lines represent the standardized mean difference (SMD) and 95% confidence intervals (CIs); the red diamond denotes the pooled effect.

In 17 studies that analyzed the histopathological parameters of colon tissue in mice, 11 measured HSC, involving a total of 132 animals. Significant heterogeneity was observed across studies (I^2^ = 59.10%, *p* = 0.007). Therefore, we employed a random-effects model to combine the results of the effect of LUT on HSC in UC models. The meta-analysis revealed that LUT intervention significantly reduced the HSC of UC model animals (SMD = −2.28, 95% CI [-3.06, −1.49], *p* < 0.001) (Figure 3A).

CL is an important indicator for assessing the severity of colonic inflammation in UC animal models. Fifteen studies investigated the effect of LUT on CL in UC animals, involving 223 animals. Given the heterogeneity across studies, we used a random-effects model to pool the effects. The meta-analysis indicated that LUT significantly increased the CL in UC animals (SMD = 2.01, 95% CI [1.13, 2.88], *p* < 0.001) (Figure 3B).

BWC reflects the overall nutritional status and can indirectly indicate how intestinal inflammation impacts the general health of UC animals. In the integrated cohort of 180 animals in 11 studies, we used a random-effects model to combine the effects of ten studies. The results demonstrated that LUT significantly improved the body weight of UC animals, suggesting an improvement in general health. However, significant heterogeneity was observed across studies (SMD = 1.98, 95% CI [0.84, 3.13], *p* < 0.001) (Figure 3C).

The DAI score summarizes the stool consistency, presence of rectal bleeding, and body weight, serving as a key indicator of intestinal inflammation. The meta-analysis of 16 studies involving 259 animals revealed that LUT intervention significantly reduced the DAI score in UC animals, highlighting its potential to alleviate intestinal inflammation (SMD = −1.68, 95% CI [-2.41, −0.96], *p* < 0.001) (Figure 3D).

#### 3.4.2 Inflammatory biomarkers

**FIGURE 4:**
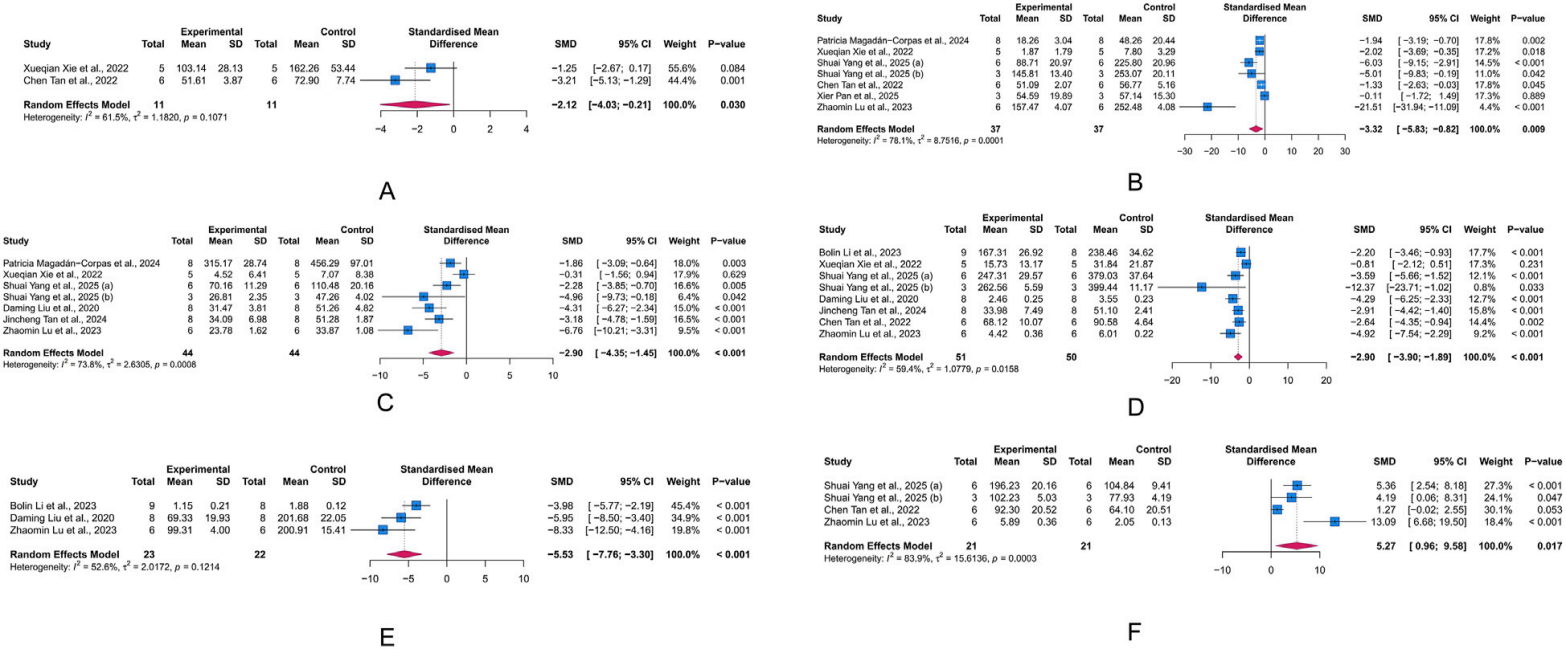
Forest plots showing results of inflammatory biomarkers. **(A)** Interleukin (IL)-17. **(B)** IL-6. **(C)** IL-1β. **(D)** Tumor necrosis factor (TNF)-α. **(E)** C-reactive protein (CRP). **(F)** IL-10. The blue squares and horizontal lines represent the standardized mean difference (SMD) and 95% confidence intervals (CIs); the red diamond denotes the pooled effect.

IL-17, IL-6, IL-1β, TNF-α, CRP, and IL-10 are major biomarkers of inflammation. At least two studies included in our analysis reported these markers. IL-1β, IL-6, and TNF-α are primary pro-inflammatory mediators, while IL-10 is a common anti-inflammatory factor. CRP, a liver-synthesized acute-phase reactant, indicates systemic inflammation. Based on a random-effects meta-analysis, LUT significantly decreased the levels of IL-17 (n = 2, SMD = −2.12, 95% CI [-4.03,-0.21], I^2^ = 61.5%, *p* = 0.03), IL-6 (n = 7, SMD = −3.32, 95% CI [-5.83, −0.82], I^2^ = 78.1%, *p* = 0.009), IL-1β (n = 7, SMD = −2.9, 95% CI [-4.35, −1.45], I^2^ = 73.8%, *p* < 0.001), TNF-α (n = 8, SMD = −2.90, 95% CI [-3.90, −1.89], I^2^ = 59.4%, *p* < 0.001), and CRP (n = 3, SMD = −5.53, 95% CI [-7.76, −3.30], I^2^ = 52.6%, *p* < 0.001), while increasing the level of IL-10 (n = 4, SMD = 5.27, 95% CI [0.96, 9.58], I^2^ = 83.9%, *p* = 0.017) (Figures 4A–F). These results demonstrate the anti-inflammatory effects of LUT in UC.

#### 3.4.3 Intestinal barrier-related parameters

**FIGURE 5:**
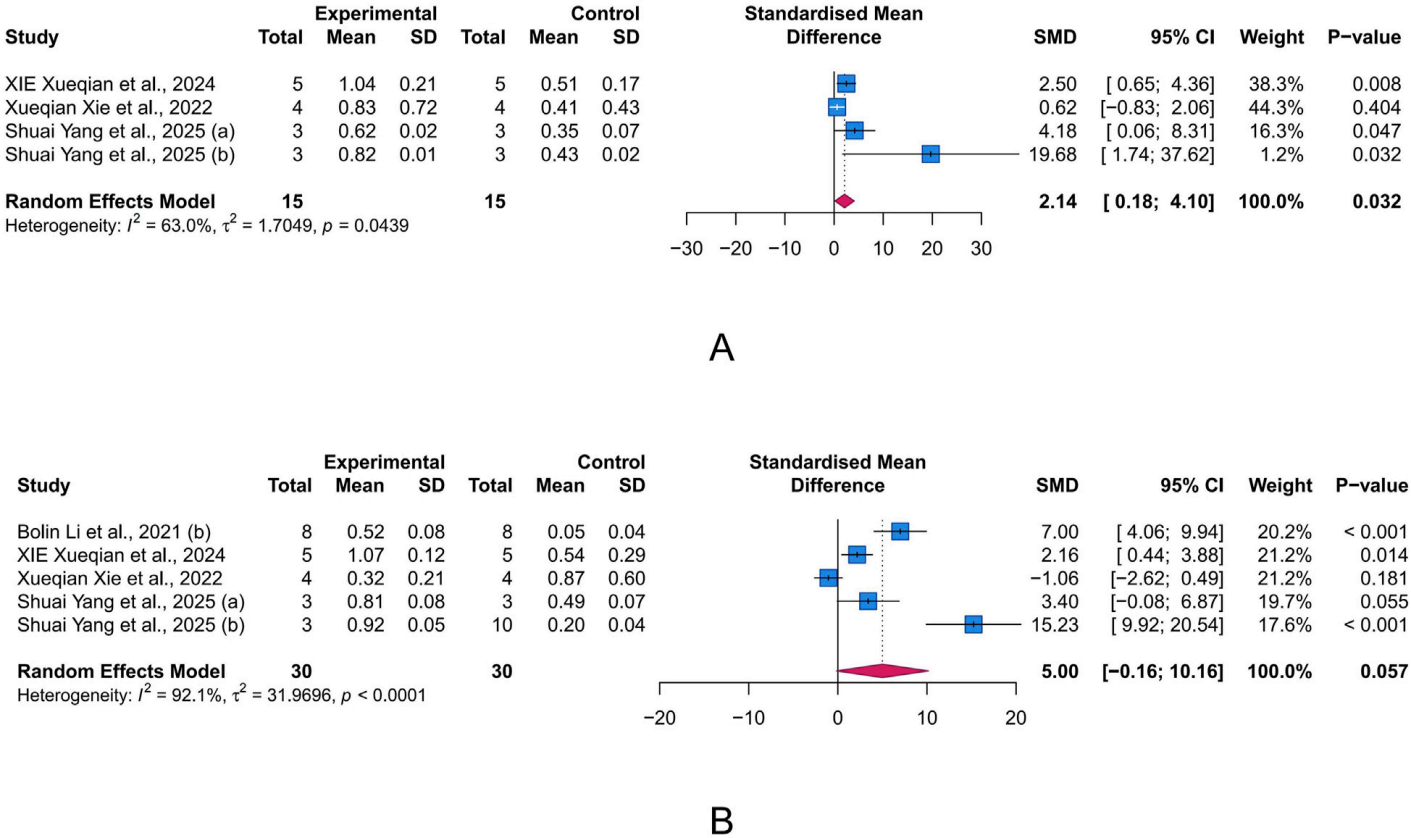
Forest plots showing results of intestinal barrier-related parameters. **(A)** occludin. **(B)** Zonula occludens-1 (ZO-1). The blue squares and horizontal lines represent the standardized mean difference (SMD) and 95% confidence intervals (CIs); the red diamond denotes the pooled effect.

Tight junctions contribute to the paracellular barrier, regulating the selective passage of substances and maintaining the integrity of intestinal barrier. Critical tight junction proteins, ZO-1 and occludin, are indicative of the integrity of this barrier. The results indicated that intervention with LUT significantly increased occludin levels in the UC animal model (n = 4, SMD = 2.14, 95% CI [0.18, 4.10], I^2^ = 63.0%, *p* = 0.032), while also showing a potential improvement effect on ZO-1 levels (n = 5, SMD = 5.00, 95% CI [-0.16, 10.16], I^2^ = 92.1%, *p* = 0.057) (Figures 5A,B). These results suggest that LUT may help restore the compromised intestinal epithelial barrier function in UC.

#### 3.4.4 Oxidative stress-related parameters

**FIGURE 6:**
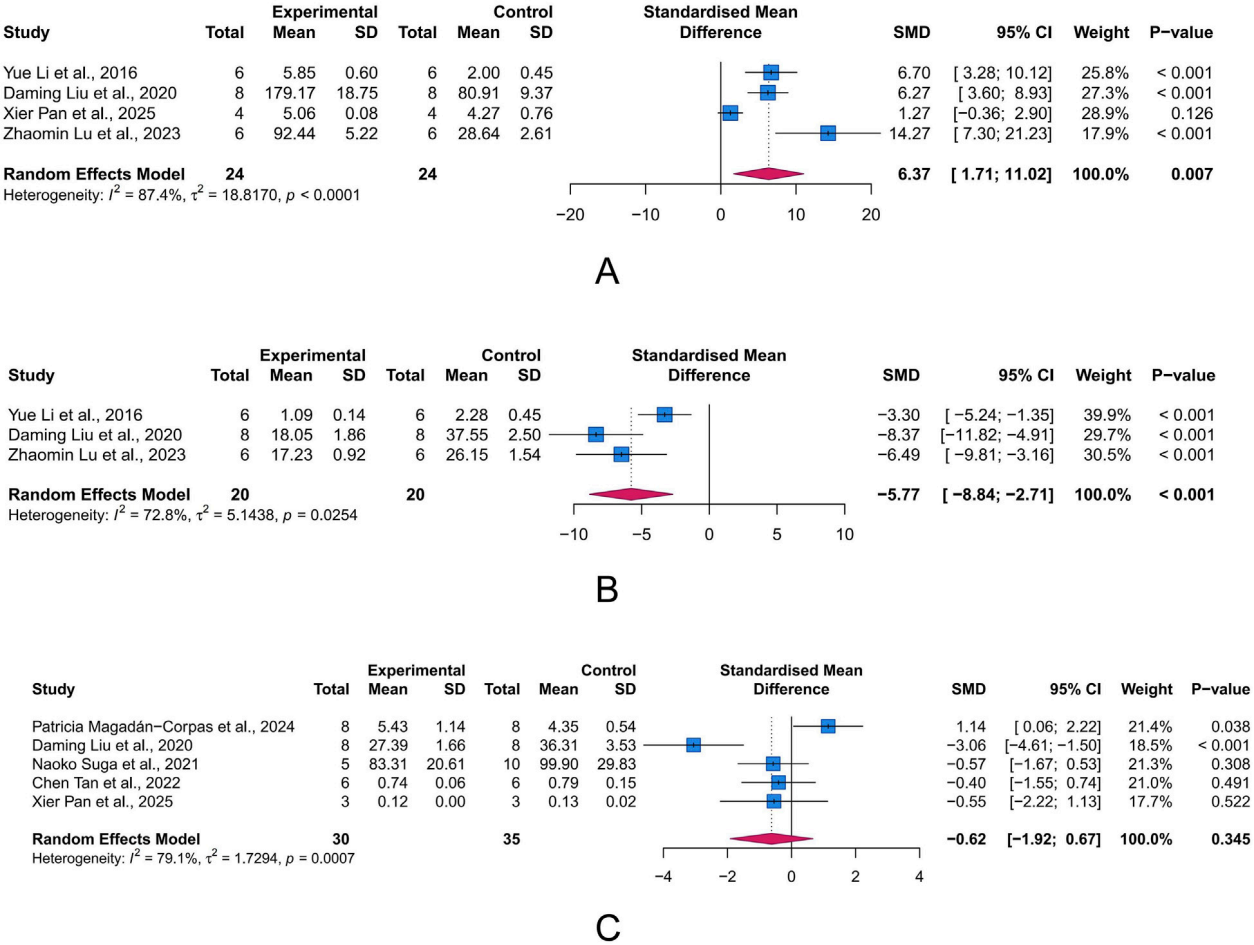
Forest plots showing results of oxidative stress-related parameters. **(A)** Superoxide dismutase (SOD). **(B)** Malondialdehyde (MDA). **(C)** Myeloperoxidase (MPO). The blue squares and horizontal lines represent the standardized mean difference (SMD) and 95% confidence intervals (CIs); the red diamond denotes the pooled effect.

Oxidative stress arises when the production of reactive oxygen species (ROS) exceeds the capacity of antioxidant defenses, leading to cell and tissue damage. Within this state, MDA (a byproduct of lipid peroxidation), SOD (a key enzyme responsible for scavenging superoxide radicals), and MPO (a pro-oxidative enzyme derived from neutrophils) serve as important biomarkers for oxidative damage, antioxidant defense, and pro-oxidant activity, respectively. We assessed the intervention effects of LUT on these three proteins to evaluate its role in UC-associated oxidative stress. The meta-analysis results showed that LUT effectively upregulated SOD levels (n = 4, SMD = 6.37, 95% CI [1.71, 11.02], I^2^ = 87.4%, *p* = 0.007) and decreased MDA levels (n = 3, SMD = −5.77, 95% CI [-8.84, −2.71], I^2^ = 72.8%, *p* < 0.001), but did not seem to affect MPO levels (n = 5, SMD = −0.62, 95% CI [-1.92, 0.67], I^2^ = 79.1%, *p* = 0.345) (Figures 6A–C). Collectively, these results demonstrate LUT’s ability to mitigate oxidative stress.

#### 3.4.5 Intestinal microbiota-related parameters

**FIGURE 7:**
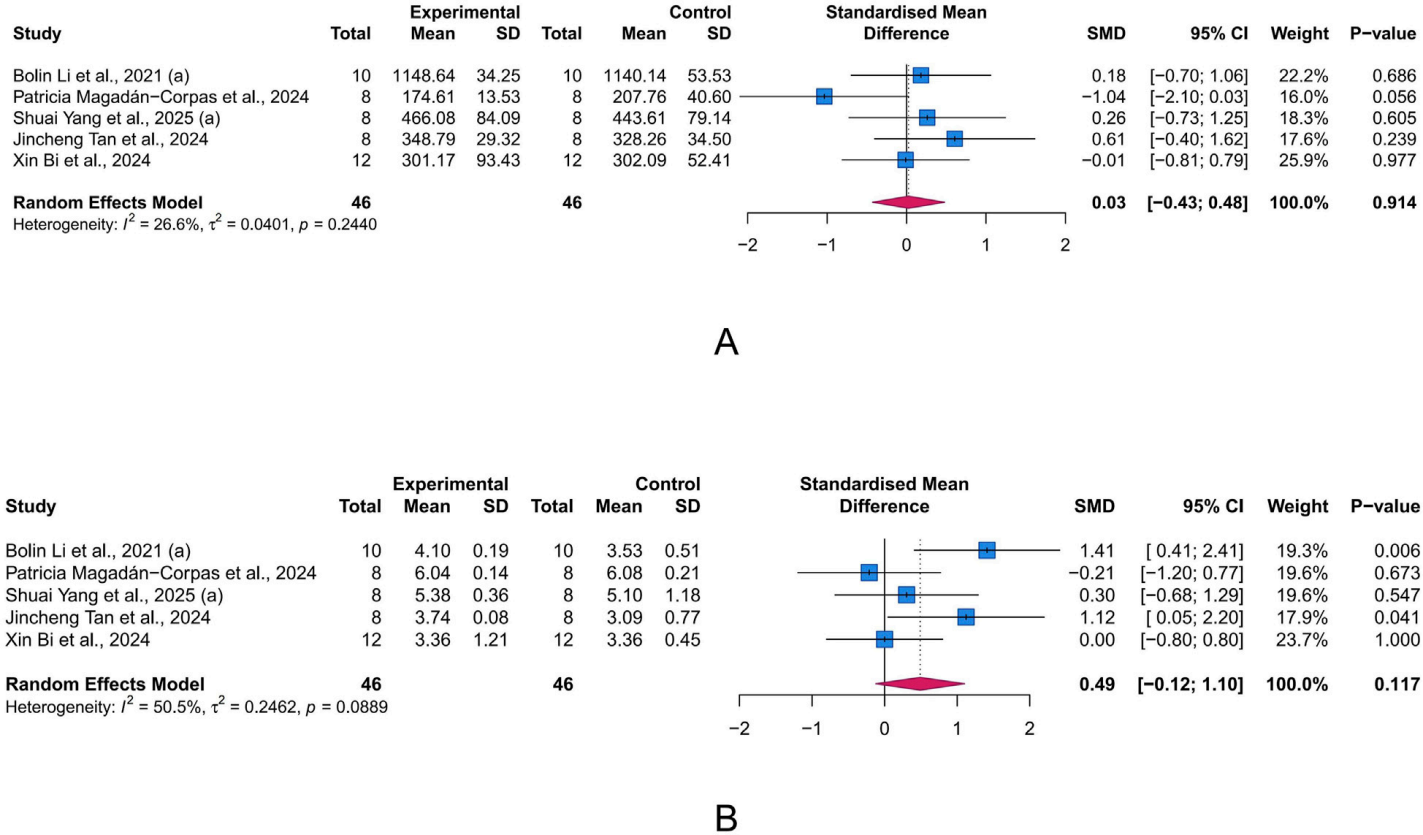
Forest plots showing intestinal microbiota-related parameters. **(A)** Chao index. **(B)** Shannon index. The blue squares and horizontal lines represent the standardized mean difference (SMD) and 95% confidence intervals (CIs); the red diamond denotes the pooled effect.

Chao index and Shannon index are commonly used α-diversity indices to measure the diversity of gut microbiota(GM). α-diversity reflects the within-sample diversity, encompassing both species richness (the number of species, as emphasized by Chao) and evenness (the relative abundance distribution, as captured by Shannon). They are key indicators of ecosystem stability and resilience, with lower values often correlating with disease severity and progression in UC. Unfortunately, according to the meta-analysis results, LUT had limited effects on the Chao index (n = 5, SMD = 0.03, 95% CI [-0.43, 0.48], I^2^ = 26.6%, *p* = 0.914) and Shannon index (n = 5, SMD = 0.49, 95% CI [-0.12, 1.10], I^2^ = 50.5%, *p* = 0.117) (Figures 7A,B).

### 3.5 Publication bias

**FIGURE 8:**
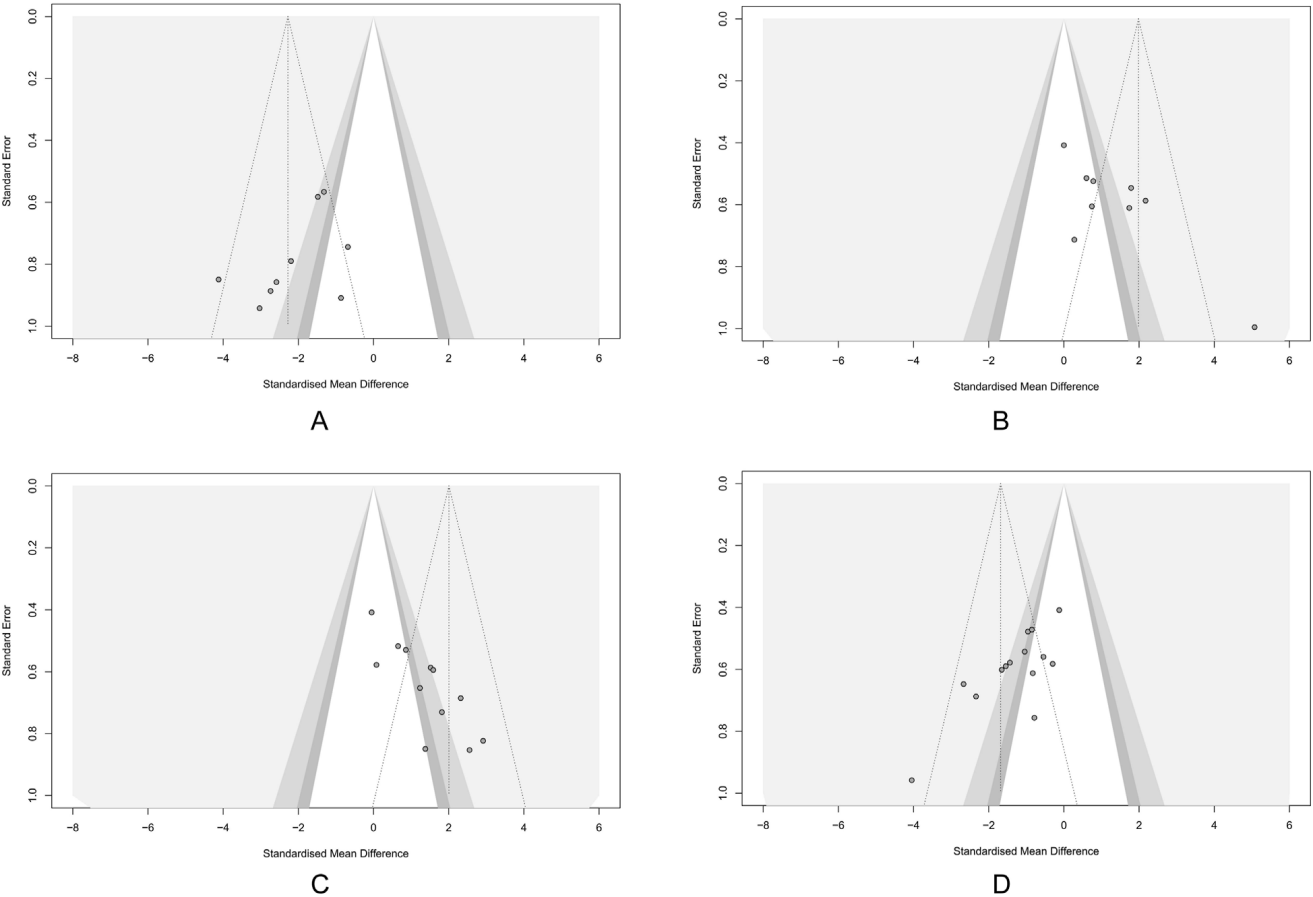
Funnel plots showing the result of publication bias. **(A)** Histological score (HSC). **(B)** Body weight change (BWC). **(C)** Colon length (CL). **(D)** Disease activity index (DAI). Each dot represents an individual study; the vertical dashed line indicates the pooled standardized mean difference (SMD), and the diagonal lines depict the 95% confidence intervals (CIs). Symmetrical distribution around the pooled effect suggests minimal publication bias; any asymmetry may indicate potential bias.

Potential publication bias was detected using funnel plots and Egger’s test in the main outcomes. The results indicated that the effect sizes of HSC, BWC, CL, and DAI studies were largely outside the funnel plot range, suggesting asymmetry (Figures 8A–D). Egger’s test revealed potential publication bias for HSC (*p* = 0.02), BWC (*p* < 0.001), CL (*p* < 0.001), and DAI (*p* < 0.001). To assess the impact of publication bias on the primary outcomes (HSC, BWC, CL, and DAI), we applied the trim-and-fill method. The results demonstrated that after correcting for publication bias with the addition of virtual studies, the effects of LUT on the primary outcomes remained robust (Supplementary Figure S1).

### 3.6 Exploring sources of heterogeneity

**FIGURE 9:**
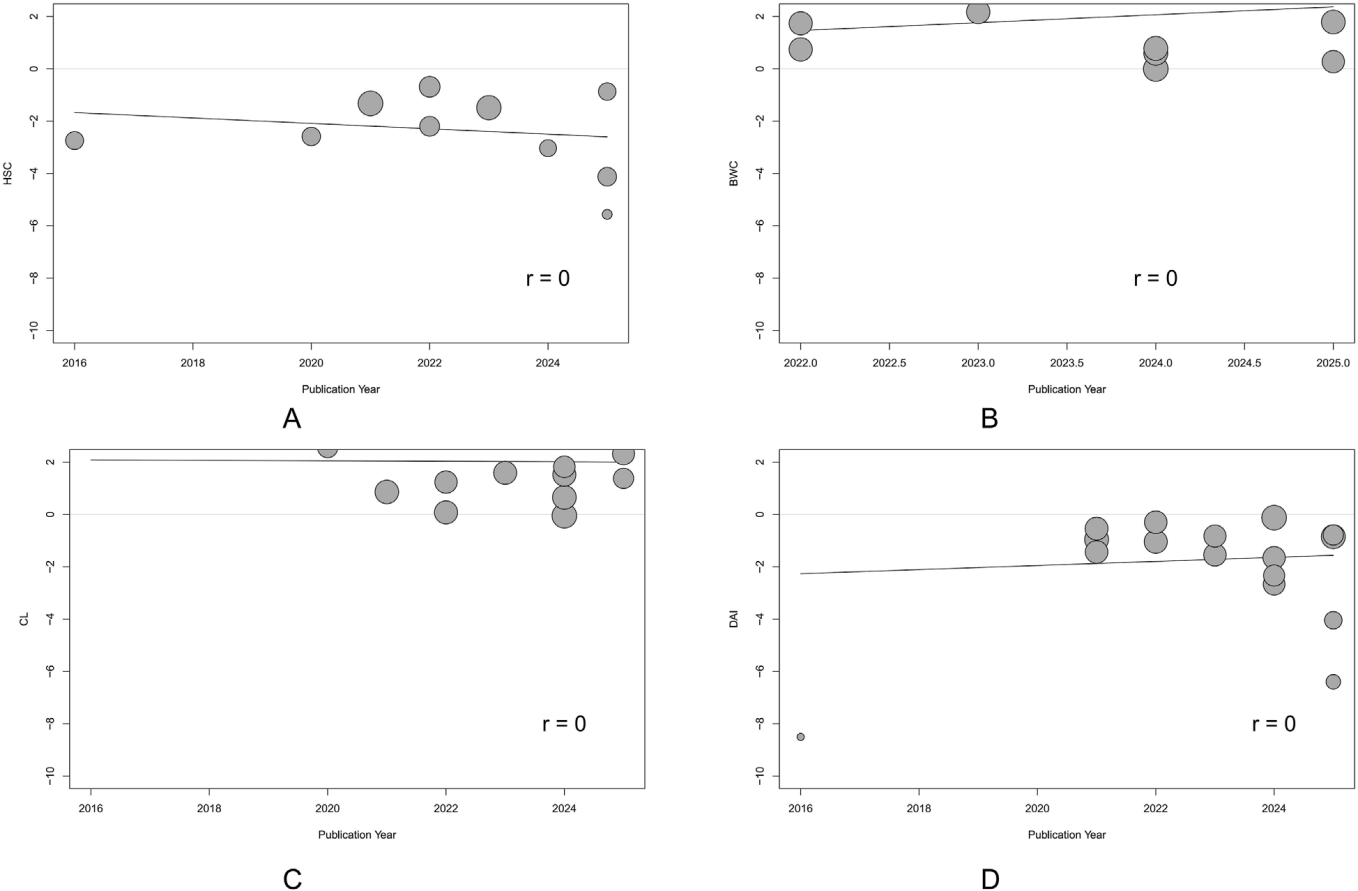
Univariate meta-regression of publication year. **(A)** Histological score (HSC). **(B)** Body weight change (BWC). **(C)** Colon length (CL). **(D)** Disease activity index (DAI). Each circle represents one study, with circle diameter proportional to its inverse-variance weight. The solid line depicts the regression slope. The non-significant slope suggests that the observed effect did not change materially over time.

**FIGURE 10:**
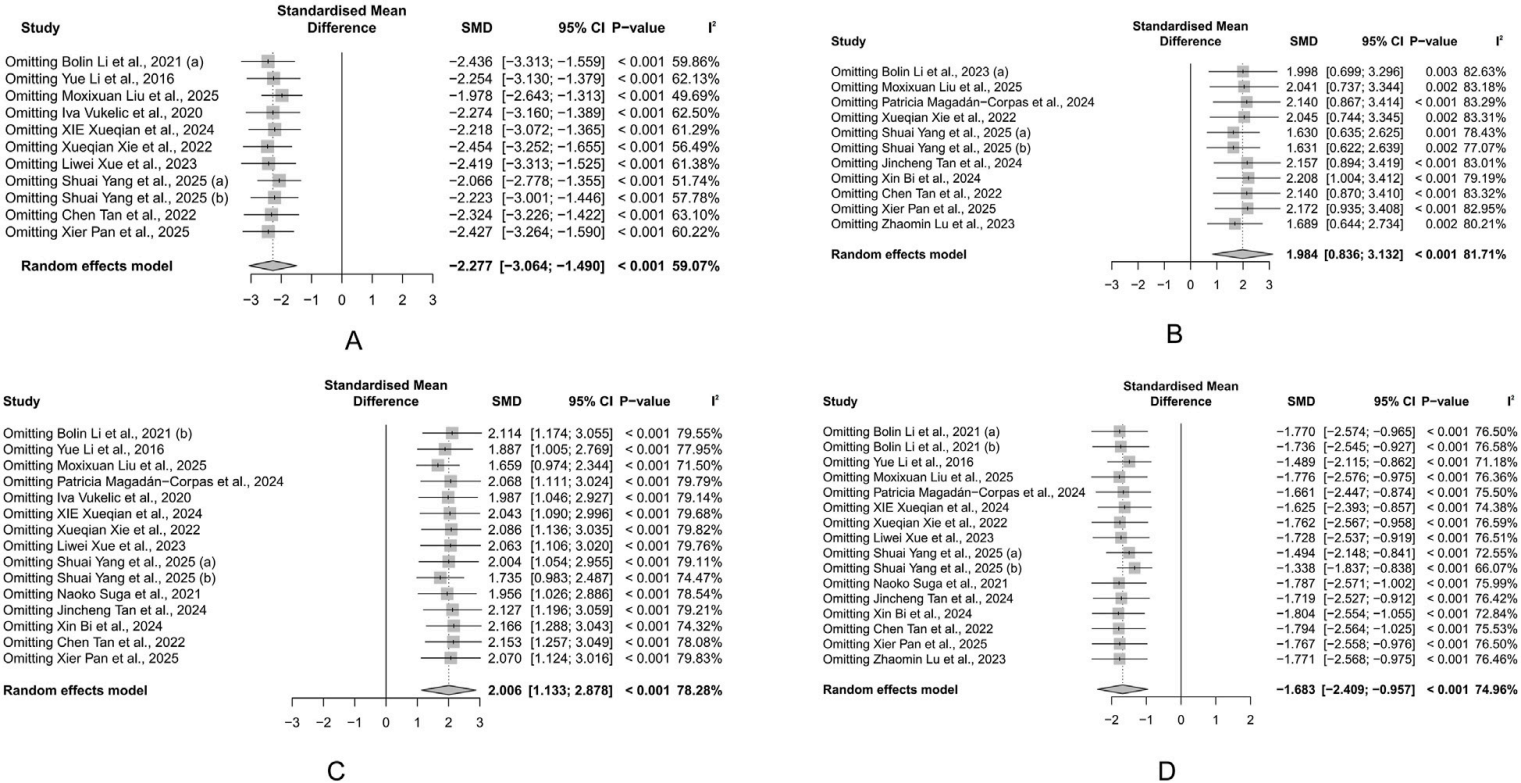
Sensitivity analysis of the study. **(A)** Histological score (HSC). **(B)** Body weight change (BWC). **(C)** Colon length (CL). **(D)** Disease activity index (DAI). Each row displays the pooled standardized mean difference (SMD) and 95% CI when the corresponding study (left column) is omitted from the meta-analysis. The vertical dashed line represents the overall SMD derived from the full dataset. The minimal fluctuation in estimates indicates that no single study disproportionately influences the overall effect.

Due to heterogeneity, we used subgroup analyses to explore potential sources. We integrated subgroup analysis, sensitivity analysis, and meta-regression to explore the sources of heterogeneity. First, we conducted subgroup analyses based on factors such as animal type, intervention duration, modeling method, and dose of administration. Subgroup analysis did not reveal any sources of heterogeneity for HSC results (Supplementary Table S2). Variations in species and modeling methods could explain the heterogeneity in DAI results, with the mouse subgroup (I^2^ = 40.3%) and the 2.5% DSS subgroup (I^2^ = 0%) showing significantly reduced heterogeneity (Supplementary Table S3). Similarly, modeling methods may also account for the heterogeneity in CL results, with the 2% DSS subgroup showing significantly reduced heterogeneity (I^2^ = 0%) (Supplementary Table S4). For BWC, treatment duration was a significant source of heterogeneity, with the subgroup of treatment durations shorter than 14 days showing significantly reduced heterogeneity (I^2^ = 0%) (Supplementary Table S5). We then conducted univariate meta-regression to explore whether publication year was a source of heterogeneity. We found that publication year could not explain the heterogeneity in any of the main outcomes (Figure 9). Lastly, we performed a sensitivity analysis to test if the results were robust. After sequentially removing each study, we found that the effects of the rest of the studies remained within the 95% CI range of the total effect, confirming the reliability of the results (Figures 10A–D).

## 4 Discussion

### 4.1 Summary of evidence

Our study suggested that LUT may alleviate inflammation and improve the function of the intestinal barrier in UC animal models, showing promise as a potential therapy for UC. Specifically, we found that LUT can increase the levels of CL, IL-10, occludin, and SOD and improve the loss of body weight. Meanwhile, we observed reductions in HSC, DAI, IL-1β, IL-6, IL-17, TNF-α, CRP, and MDA levels. Furthermore, subgroup analysis, sensitivity analysis, and meta-regression were conducted to investigate the sources of heterogeneity from six dimensions: publication year, animal type, modeling method, treatment duration, treatment dosage, and the presence of single outlier studies. The heterogeneity of DAI was from differences in animal species and modeling methods, while the heterogeneity in CL was due to variations in the modeling approach, and BWC variability could be related to treatment duration. The sensitivity analysis showed that no individual study had a substantial impact on the overall effect size, indicating that heterogeneity was not caused by any outlier study’s bias. Finally, all indicators exhibited publication bias, and the trim-and-fill method results indicated that the outcomes remained robust even after correcting for this bias.

### 4.2 Mechanism overview

#### 4.2.1 Synergistic effect of anti-oxidative stress and anti-inflammation

The literature reviewed indicates that LUT exerts inhibitory effects on various inflammatory factors, including TNF-α, IL-1β, IL-6, and IL-17, reducing their protein and mRNA expression levels (Xue et al., 2023; Yang et al., 2025a; Li et al., 2016). Furthermore, LUT has been shown to mitigate the level of CRP, which is an important biomarker in the context of UC (Yang et al., 2025a). In UC, inflammation pathways and pro-inflammatory cytokines are significantly disrupted (Voelker, 2024). The activation of immune cells, including macrophages, T helper 1(Th1), and Th17 cells, triggers the release of various pro-inflammatory cytokines, such as TNF-α, IL-1β, and IL-6 (Geremia et al., 2014). TNF-α, a key driver of intestinal inflammation, triggers a series of cellular processes that activate death signals and mitogen-activated protein kinase (MAPK) pathways, thereby enhancing the cytotoxic function of immune cells (Zhang et al., 2024). IL-1β, in conjunction with TNF-α and IL-6, can disrupt the intestinal barrier and promote immune cell activation, leading to sustained inflammation (Nakase et al., 2022). IL-17, a Th17-related cytokine, is closely related to the clinical severity of UC patients (Alexander et al., 2022). The regulatory effects of LUT on these key inflammatory factors confirm its therapeutic potential in UC. Some studies have additionally elucidated the molecular mechanisms by which LUT inhibits the release of inflammatory factors. NF-κB and proliferator-activated receptor gamma (PPAR-γ) play a role in numerous inflammation-related diseases, including UC (Althagafy et al., 2023). The included studies demonstrate that LUT treatment significantly restores NF-κB levels while elevating the suppressed PPAR-γ levels in the UC animal models (Liu et al., 2020). LUT was further found to antagonize the phosphorylation of IKKα/β and the activation of the NF-κB signaling pathway in macrophages and to inhibit C-C motif chemokine 2-induced macrophage chemotaxis, thereby inhibiting downstream inflammatory pathways (Xue et al., 2023). In addition to inhibiting macrophage-related inflammation, LUT was also believed to suppress the expression of the MAPK pathway in mast cells, thereby reducing the production of 5-hydroxytryptamine and promoting the restoration of intestinal homeostasis (Suga et al., 2021).

Oxidative stress is an important factor in promoting intestinal inflammation. ROS can activate inflammatory signaling pathways like NF-κB and MAPK pathways, which in turn stimulate the release of inflammatory mediators, contributing to dysfunction or necrosis of intestinal epithelial cells (Ca et al., 2025). MDA, the end product of lipid peroxidation, is commonly used to indicate oxidative damage (Ma et al., 2023). MPO promotes the production of ROS and chlorinated derivatives and is closely related to inflammatory response and oxidative stress. SOD is an important endogenous antioxidant that can effectively remove ROS and thus prevent oxidative damage (McCord and Edeas, 2005). The included studies showed that LUT treatment reversed the changes in these indicators in UC animal models, indicating that LUT may inhibit oxidative stress in UC (Li et al., 2016). Nucleus erythroid-related factor 2 (Nrf2), a transcription factor sensitive to redox changes, resists oxidative stress by regulating the transcription of a series of detoxification and antioxidant enzymes, and can antagonize the development of intestinal inflammation in UC (Bryan et al., 2013). Li et al. demonstrated that the antioxidant activity of LUT may be mediated by the activation of the Nrf2/HO-1 signaling pathway (Li et al., 2016). Previous studies have shown that excessive nitric oxide (NO) may induce oxidative stress and tissue necrosis (Kohli et al., 2022). LUT intervention can also inhibit the formation of overactive inducible nitric oxide synthase in UC models, thereby limiting NO formation to alleviate oxidative stress (Li et al., 2016).

#### 4.2.2 Repair of intestinal epithelial barrier

The intestinal epithelium serves as a physiological barrier, providing mechanical, chemical, biological, and immune protection, and is a key structure for maintaining intestinal homeostasis (Maloy and Powrie, 2011). The intestinal epithelium forms a selective barrier through tight junction proteins, such as ZO-1, occludin, and claudin-1, to prevent the penetration of intestinal contents (such as pathogens and toxins) (Li et al., 2022). Mucins such as Muc1, Muc2, and antimicrobial peptides have immune regulatory effects and help maintain barrier function (Johansson and Hansson, 2016). Meta-analysis of integrated cohorts showed that LUT can enhance tight junction proteins and mucin levels, exhibiting a good effect on protecting the mucosal barrier (Xue et al., 2023; Yang et al., 2025a; Liu et al., 2025).

Li et al. discovered that the role of LUT in protecting the epithelial barrier may be related to the signal transducer and activator of transcription 3 (STAT3) signaling pathway (Li B.-L. et al., 2021). Studies have shown that constitutive activation of the STAT3 signaling pathway leads to decreased tight junction protein expression (Akiyama et al., 2015), and inhibition of STAT3 activity significantly enhances intestinal mucosal barrier function (Yun et al., 2017). Recent studies have shown that Src homology region 2 domain-containing phosphatase 1(SHP-1) is involved in regulating intestinal mucosal barrier function (Fan et al., 2015). Additionally, SHP-1 can negatively regulate the activation of STAT3 by mediating Janus kinase 2 (JAK2) (Ding et al., 2021). Li et al. found that LUT can reduce the phosphorylation of STAT3 by upregulating SHP-1 (Li B.-L. et al., 2021). In addition, LUT can also restore the mucosal barrier by enhancing the expression of tight junction proteins and reducing the synergistic attack of oxidative stress and inflammation on the intestinal epithelium (Xue et al., 2023).

#### 4.2.3 Regulation of GM

Previous evidence indicates that GM is the culprit for initiating colonic inflammation, disrupting the mucosal barrier, and recruiting proinflammatory cells to infiltrate the mucosa in the early stages (López-Cauce et al., 2022). Regulating the GM helps restore intestinal immune homeostasis and relieve colonic inflammation (Luo et al., 2019). However, the integration analysis of α-diversity, as a holistic index, showed no evident changes in this study. Notably, α-diversity represents a broad measure and may be less sensitive to specific, functionally relevant shifts in microbial composition or community structure. The lack of change in α-diversity suggests that the overall richness and evenness of species within the gut community may not be the primary factor influenced by LUT in the context of UC remission. Crucially, alterations in the relative abundance of key bacterial taxa are frequently more indicative of functional changes and disease activity in UC than α-diversity alone. At the phylum level, the study by Tan et al. found that LUT intervention led to an increased proportion of Bacteroidetes, while the proportions of Firmicutes and Proteobacteria decreased (Tan et al., 2024). Specifically, LUT enhances the abundance of beneficial bacteria that generate short-chain fatty acids (SCFAs) and reduces the colonization of harmful bacteria. SCFAs primarily include acetate, propionate, and butyrate. LUT can increase the abundance of butyrate-producing strains like *Butyricicoccus*, *Ruminococcaceae*, *Lachnospiraceae*, and *Schizospiraceae* (Tan et al., 2024). Butyrate is crucial for preserving intestinal barrier integrity and exhibiting anti-inflammatory effects by regulating the differentiation of Treg cells and limiting neutrophil inflammation (Fu et al., 2013; Li G. et al., 2021). Bi et al. also reported that LUT intervention increased the content of SCFAs in the feces of UC mice (Bi et al., 2024). *Clostridium* and *Roseburia*, belonging to *Lachnospiraceae*, can improve UC by regulating bile acids and promoting the differentiation of Treg cells (Lu et al., 2025). *Ruminococcaceae*, on the other hand, can reduce the secretion of inflammatory cytokines by activating the nod-like receptor protein 3(54). Other studies have reported that LUT alters the β-diversity of the intestinal flora in UC animal models and reduces the colonization of harmful bacterial genera such as *Streptococcus*, *Staphylococcus*, *Escherichia*, and *Shigella* (Yang et al., 2025a; Magadán-Corpas et al., 2024; Tan et al., 2024). *Streptococcus* and *Staphylococcus* are closely associated with opportunistic infections in UC patients (Quaglio et al., 2022). Adherent-invasive *Escherichia coli* and *Shigella* can interact with intestinal epithelial cells and immune cells, which results in the disruption of the intestinal barrier and the stimulation of pro-inflammatory cytokine production (Li et al., 2025). However, Bi et al. found that LUT treatment did not change microbial composition compared to the model group. Therefore, the regulatory effect of LUT on the GM still needs further study (Bi et al., 2024).

#### 4.2.4 Regulation of cell metabolism

Cellular metabolism is often compromised to varying extents in UC, and enhancing the recovery of cellular energy metabolism is a key approach for repairing intestinal tissue damage (Liu et al., 2025). Impaired energy metabolism due to mitochondrial dynamics disorder is a core pathological feature of UC (Mancini et al., 2020). Disrupted mitochondrial fission can lead to excessive mitochondrial fragmentation, thereby impairing oxidative metabolism and consuming adenosine triphosphate (Westermann, 2012). Enhanced mitochondrial fission and reduced fusion were observed in the colon of UC mice and lipopolysaccharide-induced Caco-2 cells (Liu et al., 2025). LUT treatment significantly reversed this mitochondrial dynamics disorder by regulating the miR-195-5p/Notch signaling pathway and promoting healthy energy metabolism in colon cells (Liu et al., 2025). Li et al. found that LUT can improve the metabolic status of DSS-induced colitis rats, and the differential metabolites after treatment are mainly enriched in the D-glutamine metabolic pathway (Li et al., 2023). Research has indicated that glutamine is involved in inflammatory responses, oxidative stress, cellular protection, and intestinal barrier function (Fillmann et al., 2007). Jeong et al. found that glutamine exhibits anti-inflammatory effects and alleviates DSS-induced colitis by negatively regulating the MAPK signaling pathway (Jeong et al., 2018). Therefore, LUT may alleviate UC inflammation by regulating D-glutamine metabolism.

#### 4.2.5 Immune regulation

**FIGURE 11:**
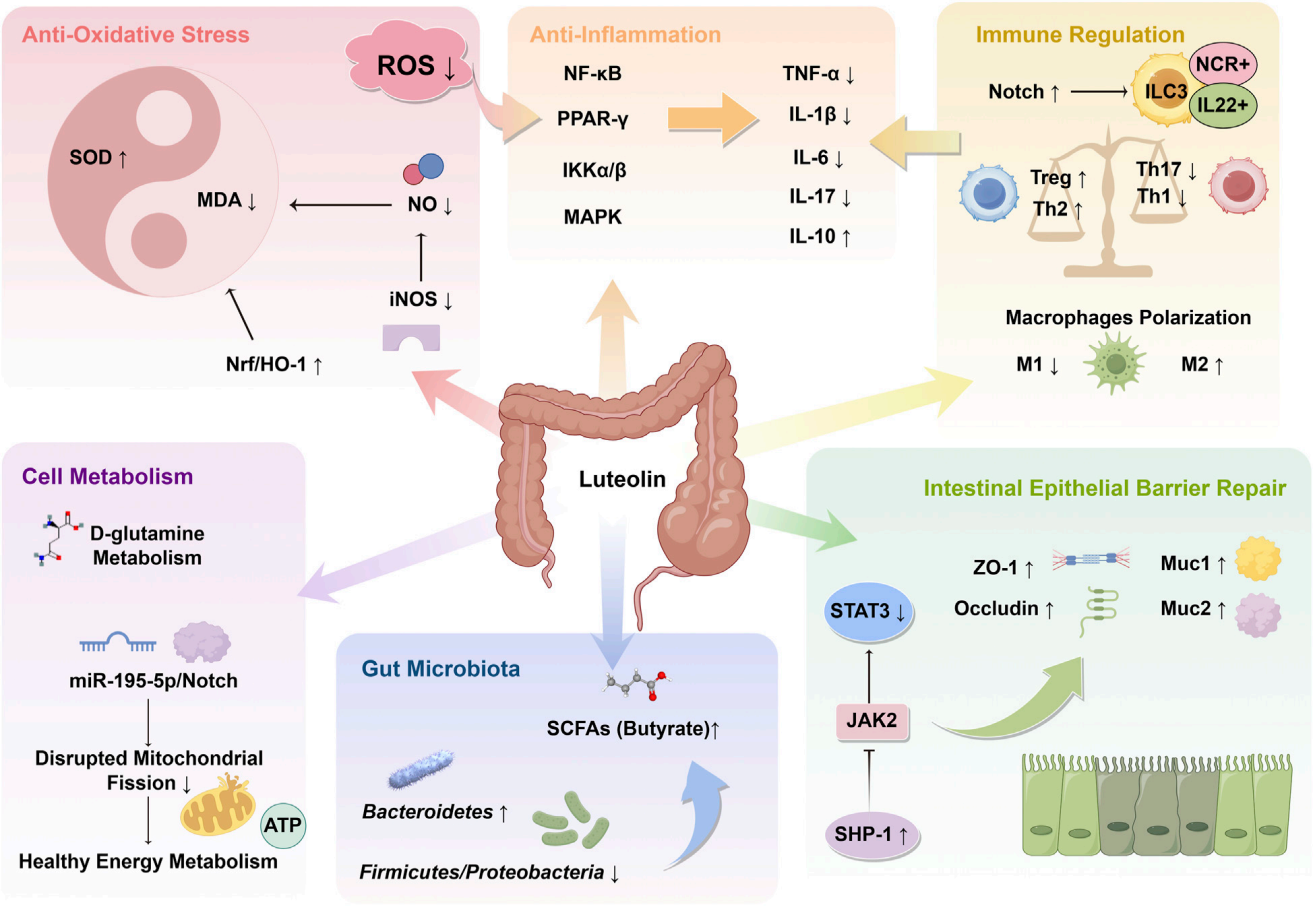
Mechanism overview of luteolin (LUT) in ulcerative colitis. LUT plays significant roles in anti-oxidative stress, anti-inflammation, immune regulation, repair of intestinal epithelial barrier, and modulation of gut microbiota and cellular metabolism. The arrows indicate the promoting or inhibitory effects of LUT. SOD, superoxide dismutase; MDA, malondialdehyde; Nrf2, nuclear factor erythroid 2-related factor 2; HO-1, heme oxygenase 1; NO, nitric oxide; iNOS, inducible nitric oxide synthase; ROS, reactive oxygen species; NF-κB, Nuclear factor-kappaB; PPAR, peroxisome proliferator-activated receptor; IKK, IkappaB kinase; MAPK, mitogen-activated protein kinase; TNF, tumor necrosis factor; IL, interleukin; ILC3, type-3 innate lymphoid cell; NCR, natural cytotoxicity receptor; SHP-1, Src homology region 2 domain-containing phosphatase 1; JAK, Janus kinase; STAT, signal transducer and activator of transcription; SCFA, short-chain fatty acid; ATP, adenosine triphosphate.

Immunodysregulation is a prominent feature of UC. During active UC, immune cells, such as Th17 cells and macrophages, significantly infiltrate the colonic tissues. In DSS-induced colitis, LUT was reported to induce macrophage polarization toward the M2 phenotype by inhibiting the expression of M1 marker genes and promoting the expression of M2 marker genes. Further transcriptomic sequencing and experimental validation revealed that the impact of LUT on macrophage polarization was regulated through the AMPK-PPARγ signaling pathway (Yang et al., 2025b). Another study found that ROS-responsive nanoparticles used for oral administration of LUT inhibited inflammation and oxidative stress in colitis mice, improving immune balance. This was characterized by an increase in Treg and Th2 cell populations, and a reduction in Th1 and Th17 cells (Tan et al., 2022). The immune modulation promoted the production of anti-inflammatory cytokines, which helped regulate the inflammatory microenvironment and resolve colonic inflammation. Moreover, group 3 innate lymphoid cells (ILC3) are also involved in gastrointestinal immune responses, which are crucial for maintaining intestinal homeostasis (Aparicio-Domingo et al., 2015). ILC3 can be categorized into NCR^+^ILC3 and NCR^−^ILC3 based on the expression of the natural cytotoxicity receptor (NCR) on their surface (Croft et al., 2022). The former secretes a large amount of IL-22, while the latter secretes substantial IL-17a. IL-22 is an important regulatory factor in maintaining epithelial homeostasis and protecting the intestinal epithelial barrier (Keir et al., 2020). Included studies have shown that LUT increases the ratio of NCR^+^ILC3 and IL-22^+^ILC3, promoting mucin secretion and enhancing the expression of ZO-1 and occludin, thereby facilitating mucosal healing (Xie et al., 2022; Xie et al., 2024). Furthermore, the modulation of ILC3 differentiation by LUT occurs through the activation of the Notch signaling pathway, and blocking the Notch pathway neutralizes the effects of LUT (Xie et al., 2024). Figure 11 briefly summarizes the mechanism of LUT in treating UC.

### 4.3 Advantages and limitations

Existing studies have demonstrated that LUT exerts an anti-inflammatory impact on various animal models and can protect intestinal health. As far as we are aware, this study represents the first systematic review and meta-analysis to explore the pharmacological effects of LUT in UC animal models using multiple metrics across different dimensions. The integrative approach helps to reduce bias and allows for the quantitative aggregation of data from multiple studies, increasing statistical power and providing more precise estimates of the effect size. It provides a rigorous and thorough understanding of the topic, offering a reliable overview of the potential mechanisms of action of LUT and supporting the translational potential of LUT-enriched diets or treatments for UC management. Notably, this review deliberately excludes *in vitro* study data, a decision with key methodological implications. Compounds like LUT, recognized as PAINS, can produce false positives due to their non-specific binding or reactivity (Bolz et al., 2021). Without thorough screening, such results may lead to exaggerated claims about the pharmacological effects of polyphenols. By omitting these studies, we eliminate potential PAINS interference and ensure that the included evidence reflects LUT’s genuine biological effects in real physiological contexts.

Nevertheless, this study has several limitations: Firstly, the studies included exhibit varying levels of quality. Key areas such as allocation concealment and blinding of the experimenters were often inadequately reported. Additionally, few studies described specific randomization methods and blinding procedures during outcome assessment. The existence of such biases could affect the overall reliability of the results. In addition, even though we performed statistical correction for publication bias using the trim-and-fill method, we must acknowledge that the existence of publication bias affected the precision of the results to some extent. This bias toward publishing positive results may lead to an overestimation of LUT’s therapeutic effects on UC. Therefore, while we have highlighted the therapeutic potential of LUT, caution is advised in interpreting these results, and additional studies are necessary to validate these findings in a more balanced context.

Secondly, although we applied pre-established subgroup analyses and meta-regression, considering factors like animal species and dosage, to reveal the sources of heterogeneity, the origins of variability remain largely unaddressed in most studies. This unexplained variation may stem from differences in experimental protocols and other detailed aspects of the studies conducted in animal models.

Thirdly, despite previous research indicating that LUT regulates GM, we have only performed a meta-analysis on the Chao index and Shannon index, and a more in-depth analysis of related indices is lacking. Furthermore, the limited number of studies included in the meta-analysis prevents a precise assessment of LUT’s effect. Future research should focus more on the regulatory effects of LUT on the GM.

Finally, while these preclinical findings strongly support LUT’s multi-target potential, translating this promise to human UC requires thorough clinical validation. Therefore, rigorously designed human trials assessing LUT’s efficacy and safety, alongside translational biomarker research to bridge mechanisms from animal models to patients, should be prioritized to fully assess its therapeutic potential in UC.

## 5 Conclusion

This study systematically evaluated the efficacy and potential mechanisms of LUT in the treatment of UC based on animal models. It suggested that LUT could alleviate the systemic manifestations and pathological scores in UC models, potentially modulating intestinal inflammation through multiple pathways. However, these findings should be interpreted with caution due to the presence of publication bias. Further clinical studies and translational research are essential to bridge the gap between animal models and human applications.

## Data Availability

The original contributions presented in the study are included in the article/Supplementary Material, further inquiries can be directed to the corresponding authors.

## References

[B1] Agrawal M. Jess T. (2022). Implications of the changing epidemiology of inflammatory bowel disease in a changing world. United Eur. Gastroenterol. J. 10, 1113–1120. 10.1002/ueg2.12317 PMC9752308 36251359

[B2] Akiyama Y. Iizuka A. Kume A. Komiyama M. Urakami K. Ashizawa T. (2015). Effect of STAT3 inhibition on the metabolic switch in a highly STAT3-activated lymphoma cell line. Cancer Genomics Proteomics 12, 133–142. 25977172

[B3] Alexander M. Ang Q. Y. Nayak R. R. Bustion A. E. Sandy M. Zhang B. (2022). Human gut bacterial metabolism drives Th17 activation and colitis. Cell Host Microbe 30, 17–30.e9. 10.1016/j.chom.2021.11.001 34822777 PMC8785648

[B4] Althagafy H. S. Ali F. E. M. Hassanein E. H. M. Mohammedsaleh Z. M. Kotb El-Sayed M. I. Atwa A. M. (2023). Canagliflozin ameliorates ulcerative colitis *via* regulation of TLR4/MAPK/NF-κB and Nrf2/PPAR-γ/SIRT1 signaling pathways. Eur. J. Pharmacol. 960, 176166. 10.1016/j.ejphar.2023.176166 37898288

[B5] Aparicio-Domingo P. Romera-Hernandez M. Karrich J. J. Cornelissen F. Papazian N. Lindenbergh-Kortleve D. J. (2015). Type 3 innate lymphoid cells maintain intestinal epithelial stem cells after tissue damage. J. Exp. Med. 212, 1783–1791. 10.1084/jem.20150318 26392223 PMC4612094

[B6] Bi X. Peng H. Xiong H. Xiao L. Zhang H. Li J. (2024). Fabrication of the rapid self-assembly hydrogels loaded with luteolin: their structural characteristics and protection effect on ulcerative colitis. Foods 13, 1105. 10.3390/foods13071105 38611409 PMC11011723

[B7] Bolz S. N. Adasme M. F. Schroeder M. (2021). Toward an understanding of pan-assay interference compounds and promiscuity: a structural perspective on binding modes. J. Chem. Inf. Model 61, 2248–2262. 10.1021/acs.jcim.0c01227 33899463

[B8] Bryan H. K. Olayanju A. Goldring C. E. Park B. K. (2013). The Nrf2 cell defence pathway: keap1-Dependent and -independent mechanisms of regulation. Biochem. Pharmacol. 85, 705–717. 10.1016/j.bcp.2012.11.016 23219527

[B9] Cao S. Lv B. Tai Y. Zuo H. X. Xing Y. Surh Y.-J. (2025). Formononetin ameliorates DSS-Induced colitis by inhibiting the MAPK/PPAR-γ/NF-κB/ROS signaling pathways. Toxicol. Appl. Pharmacol. 496, 117239. 10.1016/j.taap.2025.117239 39855309

[B10] Croft C. A. Thaller A. Marie S. Doisne J.-M. Surace L. Yang R. (2022). Notch, RORC and IL-23 signals cooperate to promote multi-lineage human innate lymphoid cell differentiation. Nat. Commun. 13, 4344. 10.1038/s41467-022-32089-3 35896601 PMC9329340

[B11] D’Haens G. R. van Deventer S. (2021). 25 years of anti-TNF treatment for inflammatory bowel disease: lessons from the past and a look to the future. Gut 70, 1396–1405. 10.1136/gutjnl-2019-320022 33431575

[B12] Ding X. Lu D. Fan J. (2021). A natural product phillygenin suppresses osteosarcoma growth and metastasis by regulating the SHP-1/JAK2/STAT3 signaling. Biosci. Biotechnol. Biochem. 85, 307–314. 10.1093/bbb/zbaa007 33604629

[B13] Fan L.-C. Shiau C.-W. Tai W.-T. Hung M.-H. Chu P.-Y. Hsieh F.-S. (2015). SHP-1 is a negative regulator of epithelial-mesenchymal transition in hepatocellular carcinoma. Oncogene 34, 5252–5263. 10.1038/onc.2014.445 25619838

[B14] Feagan B. G. Sandborn W. J. Gasink C. Jacobstein D. Lang Y. Friedman J. R. (2016). Ustekinumab as induction and maintenance therapy for crohn’s disease. N. Engl. J. Med. 375, 1946–1960. 10.1056/NEJMoa1602773 27959607

[B15] Fillmann H. Kretzmann N. A. San-Miguel B. Llesuy S. Marroni N. González-Gallego J. (2007). Glutamine inhibits over-expression of pro-inflammatory genes and down-regulates the nuclear factor kappaB pathway in an experimental model of colitis in the rat. Toxicology 236, 217–226. 10.1016/j.tox.2007.04.012 17543437

[B16] Foppa C. Rizkala T. Repici A. Hassan C. Spinelli A. (2024). Microbiota and IBD: current knowledge and future perspectives. Dig. Liver Dis. 56, 911–922. 10.1016/j.dld.2023.11.015 38008696

[B17] Furusawa Y. Obata Y. Fukuda S. Endo T. A. Nakato G. Takahashi D. (2013). Commensal microbe-derived butyrate induces the differentiation of colonic regulatory T cells. Nature 504, 446–450. 10.1038/nature12721 24226770

[B18] Geremia A. Biancheri P. Allan P. Corazza G. R. Di Sabatino A. (2014). Innate and adaptive immunity in inflammatory bowel disease. Autoimmun. Rev. 13, 3–10. 10.1016/j.autrev.2013.06.004 23774107

[B19] Hooijmans C. R. Rovers M. M. de Vries R. B. M. Leenaars M. Ritskes-Hoitinga M. Langendam M. W. (2014). SYRCLE’s risk of bias tool for animal studies. BMC Med. Res. Methodol. 14, 43. 10.1186/1471-2288-14-43 24667063 PMC4230647

[B20] Jeong S.-Y. Im Y. N. Youm J. Y. Lee H.-K. Im S.-Y. (2018). l-Glutamine attenuates DSS-induced colitis via induction of MAPK Phosphatase-1. Nutrients 10, 288. 10.3390/nu10030288 29494494 PMC5872706

[B21] Johansson M. E. V. Hansson G. C. (2016). Immunological aspects of intestinal mucus and mucins. Nat. Rev. Immunol. 16, 639–649. 10.1038/nri.2016.88 27498766 PMC6435297

[B22] Keir M. Yi T. Lu T. Ghilardi N. (2020). The role of IL-22 in intestinal health and disease. J. Exp. Med. 217, e20192195. 10.1084/jem.20192195 32997932 PMC7062536

[B23] Kohli S. K. Khanna K. Bhardwaj R. Corpas F. J. Ahmad P. (2022). Nitric oxide, salicylic acid and oxidative stress: is it a perfect equilateral triangle? Plant Physiol. Biochem. 184, 56–64. 10.1016/j.plaphy.2022.05.017 35636332

[B24] Le Berre C. Honap S. Peyrin-Biroulet L. (2023). Ulcerative colitis. Lancet 402, 571–584. 10.1016/S0140-6736(23)00966-2 37573077

[B25] Li B. Du P. Du Y. Zhao D. Cai Y. Yang Q. (2021). Luteolin alleviates inflammation and modulates gut microbiota in ulcerative colitis rats. Life Sci. 269, 119008. 10.1016/j.lfs.2020.119008 33434535

[B26] Li B. Guo Y. Jia X. Cai Y. Zhang Y. Yang Q. (2023). Luteolin alleviates ulcerative colitis in rats via regulating immune response, oxidative stress, and metabolic profiling. Open Med. (Wars) 18, 20230785. 10.1515/med-2023-0785 37693835 PMC10487402

[B27] Li B.-L. Zhao D.-Y. Du P.-L. Wang X.-T. Yang Q. Cai Y.-R. (2021). Luteolin alleviates ulcerative colitis through SHP-1/STAT3 pathway. Inflamm. Res. 70, 705–717. 10.1007/s00011-021-01468-9 34014331

[B28] Li G. Lin J. Zhang C. Gao H. Lu H. Gao X. (2021). Microbiota metabolite butyrate constrains neutrophil functions and ameliorates mucosal inflammation in inflammatory bowel disease. Gut Microbes 13, 1968257. 10.1080/19490976.2021.1968257 34494943 PMC8437544

[B29] Li H. Pan M. Li Y. Cui M. Zhang M. (2025). New targets for the treatment of ulcerative colitis: gut microbiota and its metabolites. Comput. Struct. Biotechnol. J. 27, 1850–1863. 10.1016/j.csbj.2025.05.006 40470314 PMC12136715

[B30] Li Y. Shen L. Luo H. (2016). Luteolin ameliorates dextran sulfate sodium-induced colitis in mice possibly through activation of the Nrf2 signaling pathway. Int. Immunopharmacol. 40, 24–31. 10.1016/j.intimp.2016.08.020 27569028

[B31] Li Y.-Y. Wang X.-J. Su Y.-L. Wang Q. Huang S.-W. Pan Z.-F. (2022). Baicalein ameliorates ulcerative colitis by improving intestinal epithelial barrier via AhR/IL-22 pathway in ILC3s. Acta Pharmacol. Sin. 43, 1495–1507. 10.1038/s41401-021-00781-7 34671110 PMC9160000

[B32] Liu D. Yu X. Sun H. Zhang W. Liu G. Zhu L. (2020). Flos lonicerae flavonoids attenuate experimental ulcerative colitis in rats via suppression of NF-κB signaling pathway. Naunyn Schmiedeb. Arch. Pharmacol. 393, 2481–2494. 10.1007/s00210-020-01814-4 32125461

[B33] Liu M. Wang Y. Liu Z. Liu S. Yang Q. Li B. (2025). Luteolin improves mitochondrial dynamics and function in ulcerative colitis via the miR-195-5p/Notch signalling pathway. J. Funct. Foods 124, 106644. 10.1016/j.jff.2024.106644

[B34] López-Cauce B. Puerto M. García J. J. Ponce-Alonso M. Becerra-Aparicio F. Del Campo R. (2022). Akkermansia deficiency and mucin depletion are implicated in intestinal barrier dysfunction as earlier event in the development of inflammation in interleukin-10-deficient mice. Front. Microbiol. 13, 1083884. 10.3389/fmicb.2022.1083884 36699599 PMC9869054

[B35] Lu X. Xv Y. Hu W. Sun B. Hu H. (2025). Targeting CD4+ T cells through gut microbiota: therapeutic potential of traditional Chinese medicine in inflammatory bowel disease. Front. Cell Infect. Microbiol. 15, 1557331. 10.3389/fcimb.2025.1557331 40099014 PMC11911530

[B36] Lu Z. Liu J. Zhao L. Wang C. Shi F. Li Z. (2023). Enhancement of oral bioavailability and anti-colitis effect of luteolin-loaded polymer micelles with RA (rosmarinic acid)-SS-mPEG as carrier. Drug Dev. Ind. Pharm. 49, 17–29. 10.1080/03639045.2023.2175850 36730369

[B37] Luo S. Wen R. Wang Q. Zhao Z. Nong F. Fu Y. (2019). Rhubarb peony decoction ameliorates ulcerative colitis in mice by regulating gut microbiota to restoring Th17/Treg balance. J. Ethnopharmacol. 231, 39–49. 10.1016/j.jep.2018.08.033 30170079

[B38] Ma Y. Sun W. Ye Z. Liu L. Li M. Shang J. (2023). Oxidative stress biomarker triggered multiplexed tool for auxiliary diagnosis of atherosclerosis. Sci. Adv. 9, eadh1037. 10.1126/sciadv.adh1037 37831761 PMC10575586

[B39] Magadán-Corpas P. Pérez-Valero Á. Ye S. Sordon S. Huszcza E. Popłoński J. (2024). Gut microbiota and inflammation modulation in a rat model for ulcerative colitis after the intraperitoneal administration of apigenin, luteolin, and xanthohumol. Int. J. Mol. Sci. 25, 3236. 10.3390/ijms25063236 38542210 PMC10970206

[B40] Magalhães P. R. Reis P. B. P. S. Vila-Viçosa D. Machuqueiro M. Victor B. L. (2021). Identification of pan-assay INterference compoundS (PAINS) using an MD-Based protocol. Methods Mol. Biol. 2315, 263–271. 10.1007/978-1-0716-1468-6_15 34302681

[B41] Maloy K. J. Powrie F. (2011). Intestinal homeostasis and its breakdown in inflammatory bowel disease. Nature 474, 298–306. 10.1038/nature10208 21677746

[B42] Mancini N. L. Goudie L. Xu W. Sabouny R. Rajeev S. Wang A. (2020). Perturbed mitochondrial dynamics Is a novel feature of colitis that can be targeted to lessen disease. Cell Mol. Gastroenterol. Hepatol. 10, 287–307. 10.1016/j.jcmgh.2020.04.004 32298841 PMC7327843

[B43] Mansouri P. Mansouri P. Behmard E. Najafipour S. Kouhpayeh A. Farjadfar A. (2025). Novel targets for mucosal healing in inflammatory bowel disease therapy. Int. Immunopharmacol. 144, 113544. 10.1016/j.intimp.2024.113544 39571265

[B44] McCord J. M. Edeas M. A. (2005). SOD, oxidative stress and human pathologies: a brief history and a future vision. Biomed. Pharmacother. 59, 139–142. 10.1016/j.biopha.2005.03.005 15862706

[B45] Nakase H. Sato N. Mizuno N. Ikawa Y. (2022). The influence of cytokines on the complex pathology of ulcerative colitis. Autoimmun. Rev. 21, 103017. 10.1016/j.autrev.2021.103017 34902606

[B46] Page M. J. McKenzie J. E. Bossuyt P. M. Boutron I. Hoffmann T. C. Mulrow C. D. (2021). The PRISMA 2020 statement: an updated guideline for reporting systematic reviews. BMJ 372, n71. 10.1136/bmj.n71 33782057 PMC8005924

[B47] Pan X. Xian P. Li Y. Zhao X. Zhang J. Song Y. (2025). Chemotaxis-driven hybrid liposomes recover intestinal homeostasis for targeted colitis therapy. J. Control Release 380, 829–845. 10.1016/j.jconrel.2025.02.036 39961435

[B48] Quaglio A. E. V. Grillo T. G. De Oliveira E. C. S. Di Stasi L. C. Sassaki L. Y. (2022). Gut microbiota, inflammatory bowel disease and colorectal cancer. World J. Gastroenterol. 28, 4053–4060. 10.3748/wjg.v28.i30.4053 36157114 PMC9403435

[B49] Suga N. Murakami A. Arimitsu H. Nakamura T. Nakamura Y. Kato Y. (2021). Luteolin suppresses 5-hydroxytryptamine elevation in stimulated RBL-2H3 cells and experimental colitis mice. J. Clin. Biochem. Nutr. 69, 20–27. 10.3164/jcbn.20-192 34376910 PMC8325766

[B50] Tan C. Fan H. Ding J. Han C. Guan Y. Zhu F. (2022). ROS-Responsive nanoparticles for oral delivery of luteolin and targeted therapy of ulcerative colitis by regulating pathological microenvironment. Mater Today Bio 14, 100246. 10.1016/j.mtbio.2022.100246 PMC8965165 35372817

[B51] Tan J. Wang S. Gan S. Chen H. Zhong K. Kwan H. (2024). *Centipeda minima* (L.) A. Braun and asch. and its representative active compound alleviate DSS-induced ulcerative colitis *via* inhibition of NLRP3 inflammasome activation and regulation of gut microbiota. J. Funct. Foods 117, 106207. 10.1016/j.jff.2024.106207

[B52] Voelker R. (2024). What is ulcerative colitis? JAMA 331, 716. 10.1001/jama.2023.23814 38306113

[B53] Vukelić I. Detel D. Batičić L. Potočnjak I. Domitrović R. (2020). Luteolin ameliorates experimental colitis in mice through ERK-mediated suppression of inflammation, apoptosis and autophagy. Food Chem. Toxicol. 145, 111680. 10.1016/j.fct.2020.111680 32783997

[B54] Westermann B. (2012). Bioenergetic role of mitochondrial fusion and fission. Biochim. Biophys. Acta 1817, 1833–1838. 10.1016/j.bbabio.2012.02.033 22409868

[B55] Xie X. Li P. Zhao M. Xu B. Zhang G. Wang Q. (2024). Luteolin ameliorates ulcerative colitis in mice *via* reducing the depletion of NCR+ILC3 through notch signaling pathway. Chin. J. Nat. Med. 22, 991–1002. 10.1016/S1875-5364(24)60568-6 39510641

[B56] Xie X. Zhao M. Huang S. Li P. Chen P. Luo X. (2022). Luteolin alleviates ulcerative colitis by restoring the balance of NCR-ILC3/NCR+ILC3 to repairing impaired intestinal barrier. Int. Immunopharmacol. 112, 109251. 10.1016/j.intimp.2022.109251 36182875

[B57] Xue L. Jin X. Ji T. Li R. Zhuge X. Xu F. (2023). Luteolin ameliorates DSS-induced colitis in mice via suppressing macrophage activation and chemotaxis. Int. Immunopharmacol. 124, 110996. 10.1016/j.intimp.2023.110996 37776768

[B58] Xv Y. Feng Y. Lin J. (2024). CXCR1 and CXCR2 are potential neutrophil extracellular trap-related treatment targets in ulcerative colitis: insights from Mendelian randomization, colocalization and transcriptomic analysis. Front. Immunol. 15, 1425363. 10.3389/fimmu.2024.1425363 39328405 PMC11424450

[B59] Yang S. Duan H. Yan Z. Xue C. Niu T. Cheng W. (2025a). Luteolin alleviates ulcerative colitis in mice by modulating gut microbiota and plasma metabolism. Nutrients 17, 203. 10.3390/nu17020203 39861331 PMC11768085

[B60] Yang S. Duan H. Zeng J. Yan Z. Niu T. Ma X. (2025b). Luteolin modulates macrophage phenotypic switching via the AMPK-PPARγ pathway to alleviate ulcerative colitis in mice. J. Ethnopharmacol. 339, 119157. 10.1016/j.jep.2024.119157 39603400

[B61] Yun J.-H. Park S. W. Kim K.-J. Bae J.-S. Lee E. H. Paek S. H. (2017). Endothelial STAT3 activation increases vascular leakage through downregulating tight junction proteins: implications for diabetic retinopathy. J. Cell Physiol. 232, 1123–1134. 10.1002/jcp.25575 27580405

[B62] Zhang Z. Leng Z. Kang L. Yan X. Shi J. Ji Y. (2024). Alcohol inducing macrophage M2b polarization in colitis by modulating the TRPV1-MAPK/NF-κB pathways. Phytomedicine 130, 155580. 10.1016/j.phymed.2024.155580 38810558

