# Supplementary material for: Preclinical evidence for luteolin in ulcerative colitis: a meta-analysis and systematic review
Source: Front Pharmacol. 2025 Jul 30;16:1639644. doi: 10.3389/fphar.2025.1639644 (PMC12343518; doi:10.3389/fphar.2025.1639644)
Supplement: Supplementary file 1 [file DataSheet1.docx]

**Supplementary Table 1**

Literature search strategy for LUT in the treatment of UC

| **Search Strategy (PubMed)** | |
| --- | --- |
| #1 | Idiopathic Proctocolitis [Mesh] |
| #2 | Ulcerative Colitis [Title/Abstract] |
| #3 | Colitis Gravis [Title/Abstract] |
| #4 | Inflammatory Bowel Disease, Ulcerative Colitis Type [Title/Abstract] |
| #5 | Colitis, Ulcerative [Title/Abstract] |
| #6 | #1 or #2 or #3 or #4 or #5 |
| #7 | Luteolin [Mesh] |
| #8 | 3',4',5,7-Tetrahydroxyflavone [Title/Abstract] |
| #9 | 3',4',5,7-Tetrahydroxy-Flavone [Title/Abstract] |
| #10 | Luteoline [Title/Abstract] |
| #11 | #7 or #8 or #9 or #10 |
| #12 | #6 and #11 |

**Supplementary Table 2** The subgroup analyses of HCS

|  | **Subgroup** |  | **No. of studies** | **SMD [95% CI]** | **P** | **I^2^** |
| --- | --- | --- | --- | --- | --- | --- |
| HCS | Species | mice | 11 | -2.28 [-3.06, -1.49] | <0.001 | 59.10% |
|  | treatment dose(mg/kg) | ≥50 | 6 | -2.40 [-3.42, -1.38] | < 0.001 | 60.20% |
|  |  | ＜50 | 5 | -2.20 [-3.65, -0.74] | 0.00303 | 65.60% |
|  | treatment cycles（days） | ＜14 | 6 | -2.25 [-3.16, -1.34] | <0.001 | 51.10% |
|  |  | ≥14 | 5 | -2.70 [-4.66, -0.74] | 0.0068 | 71.20% |
|  | modeling method | 4% DSS | 1 | -1.32 [-2.43, -0.21] | 0.0199 | NA |
|  |  | 3% DSS | 7 | -2.27 [-3.13, -1.41] | <0.001 | 51.30% |
|  |  | 2.5% DSS | 1 | -4.12 [-5.79, -2.46] | <0.001 | NA |
|  |  | 2%DSS | 2 | -1.78 [-4.08, 0.51] | 0.1277 | 73.80% |

**Supplementary Table 3** The subgroup analyses of DAI

|  | **Subgroup** |  | **No. of studies** | **SMD [95% CI]** | **P** | **I^2^** |
| --- | --- | --- | --- | --- | --- | --- |
| DAI | Species | rat | 3 | -1.29 [-2.12, -0.45] | 0.00253 | 40.30% |
|  |  | mice | 13 | -1.86 [-2.84, -0.89] | < 0.001 | 78.80% |
|  | treatment dose(mg/kg) | ＜50 | 8 | -1.40 [-2.25, -0.55] | 0.00122 | 73.50% |
|  |  | ≥50 | 8 | -2.24 [-3.82, -0.67] | 0.00525 | 78.60% |
|  | treatment cycles（days） | ≥14 | 10 | -1.77 [-2.76, -0.78] | < 0.001 | 78.80% |
|  |  | ＜14 | 6 | -1.45 [-2.43, -0.47] | 0.00369 | 71.40% |
|  | modeling method | 3.5% DSS | 1 | -0.96 [-1.89, -0.02] | 0.045 | NA |
|  |  | 4% DSS | 1 | -1.44 [-2.57, -0.30] | 0.01296 | NA |
|  |  | 3% DSS | 9 | -2.45 [-4.01, -0.90] | 0.00203 | 84.50% |
|  |  | 2.5% DSS | 2 | -0.72 [-1.43, -0.02] | 0.0448 | 0% |
|  |  | 2%DSS | 2 | -1.82 [-3.41, -0.23] | 0.02508 | 73% |
|  |  | 2.5%TNBS | 1 | -0.83 [-2.03, 0.37] | 0.17635 | NA |

**Supplementary Table 4** The subgroup analyses of CL

|  | **Subgroup** |  | **No. of studies** | **SMD [95% CI]** | ***P*** | **I^2^** |
| --- | --- | --- | --- | --- | --- | --- |
| CL | Species | mice | 14 | 2.07 [1.11, 3.02] | < 0.001 | 79.80% |
|  |  | rat | 1 | 1.52 [0.37, 2.67] | 0.00958 | NA% |
|  | treatment dose(mg/kg) | ≥50 | 8 | 3.34 [1.72, 4.96] | < 0.001 | 81.70% |
|  |  | ＜50 | 7 | 1.09 [0.38, 1.80] | 0.00275 | 60% |
|  | treatment cycles（days） | ≥14 | 8 | 1.83 [0.76, 2.90] | < 0.001 | 78.60% |
|  |  | ＜14 | 7 | 2.25 [0.70, 3.79] | 0.00431 | 80.70% |
|  | modeling method | 4% DSS | 1 | 0.86 [-0.18, 1.90] | 0.10434 | NA% |
|  |  | 3% DSS | 10 | 1.93 [0.82, 3.04] | < 0.001 | 79.50% |
|  |  | 2.5% DSS | 2 | 4.75 [0.92, 8.57] | 0.01492 | 84.90% |
|  |  | 2%DSS | 2 | 1.50 [0.54, 2.45] | 0.00213 | 0% |

**Supplementary Table 5** The subgroup analyses of BWC

|  | **Subgroup** |  | **No. of studies** | **SMD [95% CI]** | ***P*** | **I^2^** |
| --- | --- | --- | --- | --- | --- | --- |
| BWC | Species | rat | 3 | 2.65 [-0.08, 5.38] | 0.05683 | 81.90% |
|  |  | mice | 8 | 1.80 [0.44, 3.16] | 0.00958 | 83.30% |
|  | treatment dose(mg/kg) | ＜50 | 7 | 1.40 [0.28, 2.53] | 0.0147 | 77.60% |
|  |  | ≥50 | 4 | 3.11 [0.64, 5.58] | 0.0137 | 87.10% |
|  | treatment cycles（days） | ≥14 | 6 | 3.19 [1.22, 5.16] | 0.00153 | 89.30% |
|  |  | ＜14 | 5 | 0.88 [0.39, 1.38] | < 0.001 | 0% |
|  | modeling method | 3.5% DSS | 1 | 2.17 [1.02, 3.32] | < 0.001 | NA% |
|  |  | 2.5% DSS | 1 | 1.79 [0.72, 2.86] | 0.00106 | NA% |
|  |  | 3% DSS | 7 | 1.70 [0.06, 3.33] | 0.04197 | 84.30% |
|  |  | 2%DSS | 1 | 1.74 [0.54, 2.94] | 0.00443 | NA% |
|  |  | 2.5%TNBS | 1 | 6.01 [2.90, 9.11] | < 0.001 | NA% |


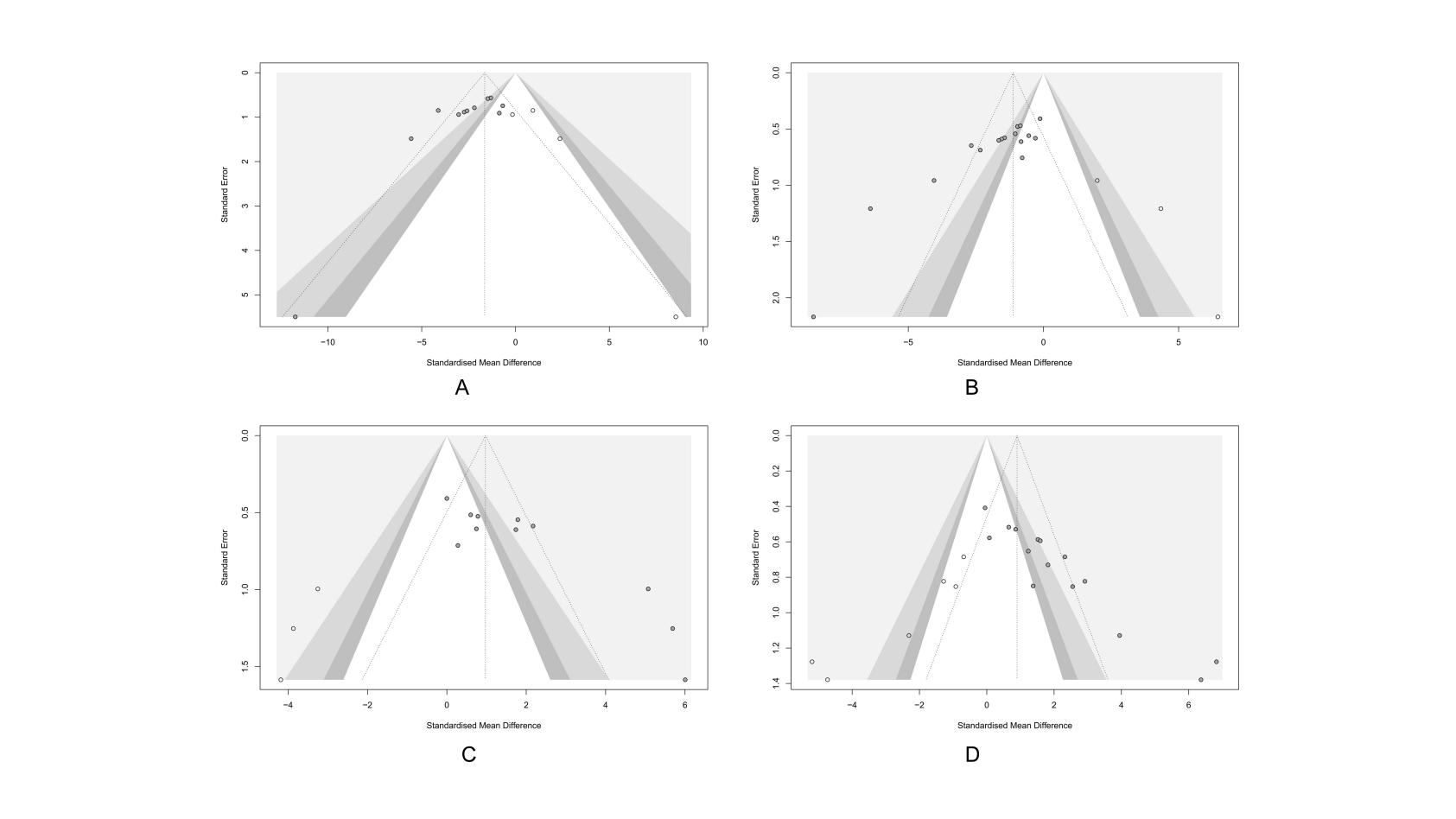


**Supplementary figure 1** The funnel plots corrected with trim-fill method
